# Missense Mutations in Exons 18–24 of EGFR in Hepatocellular Carcinoma Tissues

**DOI:** 10.1155/2015/171845

**Published:** 2015-09-07

**Authors:** Ravat Panvichian, Anchalee Tantiwetrueangdet, Pattana Sornmayura, Surasak Leelaudomlipi

**Affiliations:** ^1^Division of Medical Oncology, Department of Internal Medicine, Faculty of Medicine, Ramathibodi Hospital, Mahidol University, Bangkok 10400, Thailand; ^2^Research Center, Faculty of Medicine, Ramathibodi Hospital, Mahidol University, Bangkok 10400, Thailand; ^3^Department of Pathology, Faculty of Medicine, Ramathibodi Hospital, Mahidol University, Bangkok 10400, Thailand; ^4^Division of General Surgery, Department of Surgery, Faculty of Medicine, Ramathibodi Hospital, Mahidol University, Bangkok 10400, Thailand

## Abstract

Epidermal growth factor receptor (EGFR), a transmembrane tyrosine kinase receptor, plays important roles in various cancers. In nonsmall cell lung cancer (NSCLC), EGFR mutations cluster around the ATP-binding pocket (exons 18–21) and some of these mutations activate the kinase and induce an increased sensitivity to EGFR-tyrosine kinase inhibitors. Nevertheless, data of EGFR mutations in HCC are limited. In this study, we investigated EGFR expression by immunohistochemistry and EGFR mutations (exons 18–24) by PCR cloning and sequencing. EGFR overexpression in HCC and matched nontumor tissues were detected in 13/40 (32.5%) and 10/35 (28.6%), respectively. Moreover, missense and silent mutations were detected in 13/33 (39.4%) and 11/33 (33.3%) of HCC tissues, respectively. The thirteen different missense mutations were p.L730P, p.V742I, p.K757E, p.I780T, p.N808S, p.R831C, p.V851A, p.V897A, p.S912P, p.P937L, p.T940A, p.M947V, and p.M947T. We also found already known SNP, p.Q787Q (CAG>CAA), in 13/33 (39.4%) of HCC tissues. However, no significant association was detected between EGFR mutations and EGFR overexpression, tissue, age, sex, tumor size, AFP, HBsAg, TP53, and Ki-67. Further investigation is warranted to validate the frequency and activity of these missense mutations, as well as their roles in HCC tumorigenesis and in EGFR-targeted therapy.

## 1. Introduction

Liver cancer is the second leading cause of cancer death in men worldwide [1]. Among primary liver cancers, hepatocellular carcinoma (HCC) is the major histological subtype globally, with 78% of HCC attributable to hepatitis B virus (HBV, 53%) or hepatitis C virus (HCV, 25%) [2, 3]. Prognosis of HCC remains dismal. Owing to late diagnosis and/or advanced underlying liver cirrhosis, only limited therapeutic options with marginal clinical benefit are available for the majority of HCC patients. HCC has been considered a relatively chemotherapy refractory tumor [4]. In addition, HCC has a limited response to sorafenib, an oral multikinase inhibitor with activity against Raf-1, B-Raf, VEGFR2, PDGFR, and c-Kit receptor [5, 6]. Since sorafenib significantly increases survival of advanced-stage HCC patients when compared with placebo group (median overall survival 10.7 months versus 7.9 months and 6.5 months versus 4.2 months), the drug has been approved for the treatment of advanced-stage HCC with well-preserved liver function [7, 8]. Further understanding in HCC tumorigenesis and tumor resistant to sorafenib is needed for further development of molecularly targeted therapy in this fatal disease.

Epidermal growth factor receptor (EGFR) signaling plays an important role in various cancers, including HCC. EGFR is a 170 kDa transmembrane tyrosine kinase receptor which is activated by ligands, including epidermal growth factor (EGF) and transforming growth factor *α* (TGF-*α*). Ligand binding to the extracellular domain of EGFR results in receptor dimerization, transphosphorylation of the tyrosine residues in cytoplasmic domain, and activation of downstream signaling [9]. EGFR is highly expressed in many epithelial cancers, including lung cancer, colorectal cancer, head and neck cancer, and HCC [10–17]. EGFR overexpression is observed in 29–85% of HCC tissues [13–17]. The high percentage of EGFR overexpression in HCC has led to the suggestion of EGFR as a therapeutic target for the treatment of HCC [13].

In nonsmall cell lung cancer (NSCLC), EGFR mutations cluster around the tyrosine kinase domain (ATP-binding pocket; exons 18–21) and some of these mutations are associated with increased sensitivity to EGFR-tyrosine kinase inhibitors (EGFR TKIs) such as gefitinib, erlotinib, and afatinib; these drugs have been approved for treatment in advanced NSCLC with sensitive mutations [18–23]. Not all EGFR mutations are sensitive to EGFR TKIs; for instance, mutants at exon 20, either T790M or exon 20 insertion, are resistant while L858R and exon 19 deletion are sensitive to EGFR TKIs. NSCLC patients who have responded to EGFR TKIs and subsequently relapsed are found to have T790M secondary mutations in about 50% of cases [24–26]. In HCC, however, data of EGFR mutations in the tyrosine kinase domain are limited; only two studies have reported that no mutation was detected [27, 28]. Nevertheless, using more sensitive sequencing technique might reveal rare mutants at higher frequency. We, therefore, investigated mutation of EGFR-tyrosine kinase domain (exons 18–24) by cloning of PCR fragments obtained from RNA template of frozen HCC tissue and then sequencing. We also characterized the HCC tissues of this study by immunohistochemical staining (IHC) for EGFR protein expression, Ki-67, and tumor protein p53 (TP53) and by assays of hepatitis B antigen (HBsAg) and alpha-fetoprotein (AFP) in the serum.

## 2. Materials and Methods

This study protocol was approved by the Ethics Committee of the Faculty of Medicine, Ramathibodi Hospital, Mahidol University.

### 2.1. Liver Tissues

HCC tissues and matched nontumor tissues, that is, corresponding nontumor liver tissues available from the same patients, were obtained by surgical resections with the consent of patients.

### 2.2. Immunohistochemical (IHC) Assay

HCC tissues (*n* = 40) and matched nontumor tissues (*n* = 35) were fixed in 10% buffered formalin and then processed and embedded in paraffin. Serial 4-micron sections were cut and placed on positive charged slides. Slides were deparaffinized in xylene and hydrated through graded concentrations of ethanol and finally distilled water. Antigen retrieval was carried out at this stage with method shown in Table 1. Sections were then processed with an UltraVision LPValue Detection System (Lab Vision Corporation, CA, USA). Briefly, sections were blocked with Hydrogen Peroxide Block for 15 min at room temperature, followed by Ultra V Block for 10 min at room temperature. The following biomarkers were detected by the primary antibodies from Lab Vision Corporation: EGFR (mouse monoclonal antibody, clone 111.6); P53 (rabbit monoclonal antibody, clone Y5); and Ki-67 (rabbit monoclonal antibody, clone SP6). Primary antibody of each marker was applied at an optimized dilution and the incubation time, as shown in Table 1. Sections were incubated with Value Primary Antibody Enhancer for 30 min at room temperature; then, value HRP polymer was applied and the sections were incubated for 1 h at room temperature. DAB (3,3′-diaminobenzidine) was used as substrate to reveal the expression of each marker. Slides were counterstained with hematoxylin and mounted in permanent mounting medium. Tissues with omission of the specific antibody were used as negative controls. Slides were scanned with the Pannoramic MIDI digital slide scanner (3DHISTECH, Hungary).

### 2.3. IHC Staining Interpretation

EGFR protein expression was scored as follows: 0: no staining; 1+: weak and incomplete membranous staining in >10% of the tumor cells; 2+: weak to moderate, complete membranous staining in >10% of the tumor cells; 3+: strong, complete membranous staining in >30% of the tumor cells. Score 0 and score 1+ were determined as negative, whereas scores 2+ and 3+ were determined as positive (overexpression).

Tumor protein p53 (TP53) positivity was defined as a p53 staining with positive nuclei in ≥1% of cells, regardless of intensity of staining. Ki-67 proliferation index was assessed by visual estimation of the percentage of positive cells. Ki-67 proliferation index ≥10% was defined as Ki-67 positive by immunohistochemistry (<10% as negative; 10–19% as mildly positive, 20–29% as moderately positive, and >30% as strongly positive).

### 2.4. Serum Hepatitis B Surface Antigen (HBsAg) Assay

Chemiluminescent microparticle immunoassays (CMIA) for the qualitative detection of hepatitis B surface antigen (HBsAg) in sera from the patients were performed using ARCHITECT HBsAg Qualitative II assay (Abbot Laboratories, Illinois, USA). HBsAg negative (nonreactive) or HBsAg positive (reactive) in the serum was determined by comparing the chemiluminescent signal in the serum to the cutoff signal derived from an active calibration (according to the assay protocol, serum/cutoff signal ratio < 1.00 = nonreactive, ≥1.00 = reactive).

### 2.5. Serum Alpha-Fetoprotein (AFP) Assay

Electrochemiluminescence immunoassays (ECLIA) for the in vitro quantitative determination of alpha-fetoprotein (AFP) in sera from the patients were performed using the AFP kit with a cobas e601 analyzer (Roche Diagnostics Limited GmbH, Mannheim, GM). The serum AFP levels of the patients were collected and classified as <500 ng/mL or ≥500 ng/mL.

### 2.6. Mutation Analysis

HCC tissues from 33 HCC patients obtained by surgical resection were snap-frozen at −80°C. Briefly, tissue blocks with cryoembedding media were prepared and stored frozen at −80°C until being used. Afterward frozen tissue sections were prepared and stained with hematoxylin and eosin (H&E). The pathologist used the H&E slides for mapping the tumor areas in the frozen tissue blocks. Then RNA was isolated from these mapped tumor areas in the frozen tissues block using RNeasy Mini kit (Qiagen, Valencia, CA, USA) according to the manufacturer's protocol. Cloning and sequencing of reverse transcription polymerase chain reaction (RT-PCR) amplicons were carried out to analyze subtle mutations. First-strand cDNA synthesis was performed by using Iscript cDNA synthesis kit (Bio-Rad, USA). Specific PCR primers were used to amplify the EGFR gene (exons 18–24), forward primer 5′ CGTTCGGCACGGTGTATAA 3′ and reverse primer 5′ GGCGACTATCTGCGTCTATCAT 3′. Thermal cycling conditions involved an initial denaturation step for 5 min at 94°C, followed by 35 cycles of 30 sec at 94°C, 30 sec at 58°C, and 60 sec at 72°C, with a final extension step at 72°C for 10 min. PCR products were electrophoresed on an agarose gel to ensure that correctly sized amplicons were obtained. Amplicons were purified with QIAquick PCR Purification kit (Qiagen, Valencia, CA, USA). Purified amplicon was subcloned into pPrime cloning vector (5 PRIME, Gaithersburg, USA) according to the manufacture's protocol. Colony PCR was used for positive colony screening. The same specific primer and thermocycling condition as above were used. Then, the positive colonies were cultured in 5 mL LB with ampicillin (100 *μ*g/mL) at 37°C for 16–18 h. Plasmids were prepared by QIAprep Spin Miniprep Kit (Qiagen, Valencia, CA, USA) according to the manufacturer's protocol. The sequences were analyzed by ABI PRISM 3730XL (Applied Biosystem, USA) bidirectional using T7 promoter and SP6 primer at Macrogen. Sequences of PCR amplicons were compared with the cDNA sequence of EGFR obtained from Genbank (accession number NM 005228.3).

### 2.7. Statistical Analysis

Statistical analyses were performed with SPSS v.11.5 (SPSS Inc., Chicago, Illinois, USA). Association between EGFR overexpression or EGFR mutation and other clinicopathological variables was determined using a chi-square (*χ* ^2^) test. The values less than 0.05 were considered statistically significant.

## 3. Results

### 3.1. EGFR IHC Analysis

IHC analysis was performed in 40 HCC and 35 matched noncancerous tissues. The clinicopathological features of the patients are illustrated in Table 2. In HCC tissues, EGFR overexpression was detected in 13/40 (32.5%) tissues. Of these thirteen tissues, EGFR overexpression was scored as 3+ and 2+ in 6 and 7 tissues, respectively. However, in matched noncancerous tissues, we also detected EGFR overexpression in 10/35 (28.6%) tissues. Of these ten tissues, EGFR overexpression was scored as 3+ and 2+ in 2 and 8 tissues, respectively. Moreover, there were 5 cases which showed EGFR overexpression (score 2+) only in matched noncancerous tissues. No significant association was detected between EGFR overexpression and tissues (matched noncancerous liver and HCC), age, sex, tumor size, AFP, HBsAg, TP53 as well as Ki-67 (as shown in Supplementary data Table 1 (see Supplementary Material available online at http://dx.doi.org/10.1155/2015/171845)).

### 3.2. EGFR Mutation Analysis

**Figure 1:**
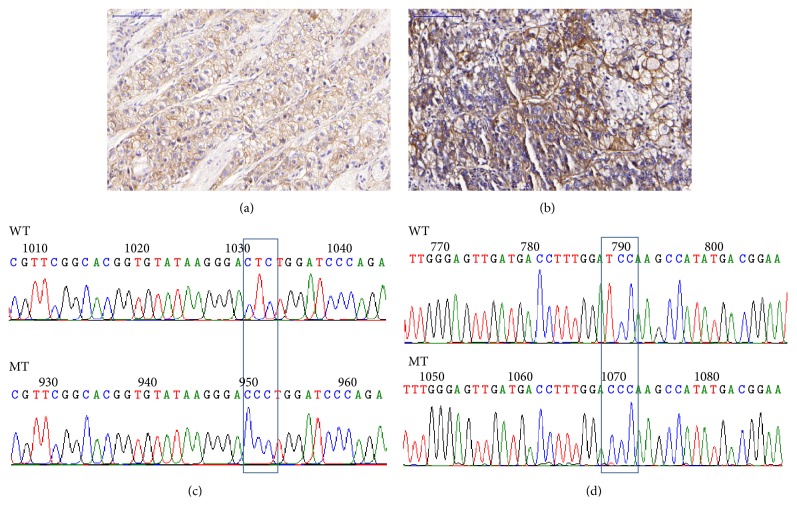
Representative of EGFR immunohistochemistry staining and electropherogram. (a) IHC score as 2+ in HCC tissue: case 6T. (b) IHC score as 3+ in HCC tissue: case 45T. (c) Missense mutation at c.2189T>C (p.L730P) in HCC tissue: case 6T. (d) Missense mutation at c.2734T>C (p.S912P) in HCC tissue: case 45T.

Only 33 frozen HCC tissues from the 40 HCC tissues in which EGFR IHC had been analyzed were available for EGFR mutation analysis. We investigated mutation of EGFR from exon 18 to exon 24. No mutation was detected in exon 18 and exon 24. However, missense and silent mutations were detected in exons 19–23. Missense and silent mutations were detected in 13/33 (39.4%) and 11/33 (33.3%) of HCC tissues, respectively. Thirteen different missense mutations were found, as shown in Table 3. Each missense mutation was found only in one (3.03%) of the HCC tissues. In addition, we found 3 missense mutations in case number 30T (p.N808S, p.R831C, and p.V897A). The representative of EGFR staining and the corresponding electropherogram of missense mutation in HCC are shown in Figure 1. In addition, we also found eleven silent mutations, as shown in Table 4. Silent mutation p.E762E was found in 2/33 (6.06%) of HCC tissues (case number 26T and case number 48T), while other silent mutations were found only in one of the HCC tissues. We also detected already known SNP, p.Q787Q (CAG>CAA), in 13/33 (39.39%) of the HCC tissues. Both missense and silent mutations were not significantly associated with age, sex, tumor size, AFP, HBsAg, TP53, Ki-67, and EGFR overexpression (as shown in supplement data Table 2).

## 4. Discussion

EGFR overexpression is detected in many epithelial cancers including lung cancer, colorectal cancer, head and neck cancer, and HCC [10–17]. EGFR overexpression has been observed in 29–85% of HCC tissues [13–17]. In this study, EGFR overexpression was detected in both HCC (32.5%) and matched nontumor tissues (28.6%). In addition, there were 5 cases which showed EGFR overexpression (score 2+) only in matched noncancerous tissues. The EGFR expression present in some HCC and not in others, demonstrated in this study, may reflect the finding of DeCicco et al. who noted that while early lesions of HCC display EGFR overexpression, advanced and differentiated HCCs tend to lose their EGFR overexpression [29]. In addition, Ito et al. have reported that EGFR expression in HCC is correlated with the proliferating activity, stage, intrahepatic spread, extrahepatic metastasis, and recurrence [15]. However, in the present study, there was no significant association between EGFR overexpression in HCC tissues and age, sex, tumor size, AFP, HBsAg, TP53, and Ki-67; this might arise from a relatively small sample size of the present study; therefore a larger confirmatory study is needed.

EGFR expression in NSCLC as detected by IHC is not an effective predictor of response to EGFR TKIs. The most specific predictive marker in NSCLC for TKI response is EGFR mutation in the tyrosine kinase domain, which has been reported in 3–32% of the lung carcinomas [18–20]. TKIs compete with ATP in binding to the ATP-binding pocket (catalytic domain of the kinase), which in turn inhibits EGFR transphosphorylation and downstream signaling.

There are different techniques used to identify the sequences of gene product with difference sensitivity and application. The sensitivity of Sanger direct sequencing is suboptimal for clinical tumor samples. However, Sanger direct sequencing is available in most molecular diagnostic laboratories and it has the advantage of detecting alterations across a gene, including novel variants. In a heterogeneous tumor, there must be at least 10–30% mutant DNA in the background of normal DNA. Therefore, using RNA instead of genomic DNA as the source for EGFR sequencing could minimize the influence of nontumor cells [30]. In this study, we used RNA as a template in order to enrich mutant EGFR from tumor cells. In addition, to alleviate the effect of tissue fixation, all of tissue samples in this study were snap frozen tissues.

Thirteen different missense mutations were detected in this study and all of these mutations have never been reported. Three missense mutations (p.L730P, p.V742I, and p.K757E) were located in exon 19, two missense mutations (p.I780T, pN808S) were located in exon 20, two missense mutations (p.R831C, p.V851A) were located in exon 21, one missense mutation (p.V897A) was located in exon 22, and five missense mutations (p.S912P, p.P937L, p.T940A, p.M947V, and p.M947T) were located in exon 23. We note that most of missense mutations in HCC clustered in exon 23, in contrast to NSCLC. In case 45T, missense mutation at S912P of exon 23 and EGFR overexpression (score 3+) were detected in HCC tissue. In addition, case 56T with missense mutation at p.P937L of exon 23 showed EGFR overexpression (score 2+) in HCC tissue. However, EGFR overexpression was not detected in cases that showed p.T940A, p.M947V, and p.M947T missense mutations of exon 23. For missense mutations in other exons, only case 6T with missense mutation at p.L730P of exon 19 showed EGFR overexpression (score 2+) in HCC tissue. In case 30T, 5 clones were selected for sequencing and three missense mutations were found at p.N808S, p.R831C, and p.V897A in exons 20, 21, and 22, respectively. Of these 5 clones, three showed p.Q787Q, one showed p.N808S and p.V897A, and one showed p.R831C and p.Q787Q. From these results, we observed that more than one mutation can be detected in each HCC tissue. In this study, we also could find already known SNP in exon 20 (p.Q787Q) at high frequency (39.39%); this SNP has been described in NSCLC and head and neck cancer [31, 32]. Furthermore, eleven silent mutations were also detected, especially p.E762E which could be found in 2 (6%) of the HCC tissues. No significant association between EGFR overexpression and missense or silent mutation was detected in this study.

Until now, no previous report has demonstrated EGFR missense mutation in HCC. Su et al. investigated the kinase domain (exons 18–21) of the EGFR gene by direct sequencing in 89 HCC tissues. They found that no missense mutation was detected in HCC tissues. However, two silent mutations (p.Y764Y, p.V819V) and a known SNP (p.Q787Q) were detected [27]. Lee et al. also studied exons 18–21 of the EGFR from 100 HCC tissues and found no exonic mutation [28]. By comparison, Su et al. and Lee et al. chose to identify sequence of the PCR products directly while we chose to identify sequence of the PCR products after cloning due to its competency to identify minority point mutations. Nevertheless, both approaches have different advantages and disadvantages.

DNA cloning is a powerful simple method for purifying a particular DNA fragment from a complex mixture of fragments and producing large numbers of the fragments of interest. Hence, sequencing after DNA cloning helps to unveil the minority point mutations (i.e., a few mutants within a high excess of wild-type alleles). However, PCR protocols occasionally generate misincorporation of nucleotides by the DNA polymerase during PCR amplification. Consequently, cloning those misincorporated PCR products and then sequencing would display the PCR artefact/mutations in some clones. Therefore, post-PCR cloning and then sequencing have the advantage in competency to identify the minority point mutations in the original template but also have the disadvantage due to displaying the PCR artefact.

When PCR products are used directly for sequencing, the consensus sequence of the amplicon will be obtained in spite of the error during PCR. This is because the errors are distributed randomly; therefore, for every molecule that has an error at a particular nucleotide position, there will be many molecules having the correct base at that position. However, in order to have good quality sequencing data, there must be at least 10–30% mutant DNA in the background of normal DNA; this requirement could be problematic. Therefore, sequencing of PCR product directly (after gel purification) has advantage in detection of the consensus sequence of the majority population in the amplicon but has the disadvantage in competency to detect the minority point mutations.

In conclusion, by using RNA template from frozen tissues, cloning PCR products of EGFR (exons 18–24) and then sequencing, we were able to detect 13 novel missense and 11 silent mutations in HCC tissues. It is still unknown whether these missense mutations play a role in HCC tumorigenesis. However, no significant association was detected between missense or silent mutations and other variables (age, sex, tumor size, AFP, HBsAg, TP53, Ki-67, and EGFR overexpression). Further investigation is warranted to validate the frequency and activity of these missense mutations, as well as their roles in HCC tumorigenesis and in EGFR-targeted therapy.

## Supplementary Material

Table 1. Association between EGFR overexpression and other variables in HCC tissues.
Table 2. Association between EGFR mutation and other variables in HCC tissues.
Electropherograms of the 13 missense mutations in EGFR exons 19–23 detected in hepatocellular carcinoma tissues.
Electropherograms of the 11 silent mutations in EGFR exons 19–23 detected in hepatocellular carcinoma tissues.

## Figures and Tables

**Table 1:** Antibodies, dilution, antigen retrieval method, and incubation time of different biomarkers.

Marker	Antibody	Dilution	Antigen retrieval method	Incubation time
EGFR	Mouse monoclonal antibody, clone 111.6, Lab Vision	1 : 400	Proteinase XXV (1 mg/mL PBS), 15 min at 37°C	2 h, RT
P53	Rabbit monoclonal antibody, clone Y5, Lab Vision	1 : 100	10 mM citrate buffer pH 6.0, pressure cooker	30 min, RT
Ki-67	Rabbit monoclonal antibody, clone SP6, Lab Vision	1 : 200	10 mM citrate buffer pH 6.0, pressure cooker	30 min, RT

**Table 2:** Clinicopathological features of HCC patients which their tissue were determined by IHC.

Patients (*n* = 40)	*n* (%)
Age (years)	
<50	19 (47.5)
≥50	21 (52.5)
Range	35–94
Mean	51.6
Median	50.5
Sex	
Male	35 (87.5)
Female	5 (12.5)
HBsAg	
Negative	11 (27.5)
Positive	29 (72.5)
AFP	
<500 ng/mL	24 (60)
≥500 ng/mL	14 (35)
Unknown	2 (5)
Tumor size	
<5 cm	13 (32.5)
≥5 cm	27 (67.5)
TP53 expression	
Negative	20 (50)
Positive	20 (50)
Ki-67 expression	
Negative (10%)	9 (22.5)
Positive (≥10%)	31 (77.5)

HBsAg: hepatitis B surface antigen; AFP: alpha-fetoprotein; HCC: hepatocellular carcinoma; TP53: tumor protein p53.

**Table 3:** Thirteen missense EGFR mutations in exons 19–23 were detected in hepatocellular carcinoma tissues.

Number	Case number	Exon	Nucleotide change	Amino acid change
1	6T	19	c.2189T>C	p.L730P
2	2T	19	c.2224G>A	p.V742I
3	17T	19	c.2269A>G	p.K757E
4	3T	20	c.2339T>C	p.I780T
5	30T	20	c.2423A>G	p.N808S
6	30T	21	c.2491C>T	p.R831C
7	15T	21	c.2552T>C	p.V851A
8	30T	22	c.2690T>C	p.V897A
9	45T	23	c.2734T>C	p.S912P
10	56T	23	c.2810C>T	p.P937L
11	36T	23	c.2818A>G	p.T940A
12	5T	23	c.2839A>G	p.M947V
13	41T	23	c.2840T>C	p.M947T

**Table 4:** Eleven silent EGFR mutations in exons 19–23 were detected in hepatocellular carcinoma tissues.

Number	Case number	Exon	Nucleotide change	Amino acid
1	36T	19	c.2199A>G	p.P733P
2	41T	19	c.2202A>G	p.E734E
3	10T	19	c.2250A>G	p.A750A
4	56T	19	c.2262A>G	p.K754K
5	26T, 48T	20	c.2286A>G	p.E762E
6	32T	21	c.2523G>A	p.R841R
7	8T	21	c.2544G>A	p.P848P
8	20T	22	c.2634C>T	p.I878I
9	5T	22	c.2673T>C	p.Y891Y
10	6T	23	c.2757T>C	p.P919P
11	45T	23	c.2793A>G	p.E931E

## Abbreviations

AFP: Alpha-fetoprotein
EGF: Epithelial growth factor
EGFR: Epithelial growth factor receptor
HBsAg: Hepatitis B surface antigen
HBV: Hepatitis B virus
HCC: Hepatocellular carcinoma
HCV: Hepatitis C virus
H&E: Hematoxylin and eosin
IHC: Immunohistochemical staining
Ki-67: Ki-67 antigen
mRNA: Messenger RNA
TKI: Tyrosine kinase inhibitor
TP53: Tumor protein p53.
