# Supplementary material for: Missense Mutations in Exons 18–24 of EGFR in Hepatocellular Carcinoma Tissues
Source: Biomed Res Int. 2015 Sep 7;2015:171845. doi: 10.1155/2015/171845 (PMC4575985; doi:10.1155/2015/171845)
Supplement: Supplementary file 1 — Table 1. Association between EGFR overexpression and other variables in HCC tissues. Table 2. Association between EGFR mutation and other variables in HCC tissues. Electropherograms of the 13 missense mutations in EGFR exons 19–23 detected in hepatocellular carcinoma tissues. Electropherograms of the 11 silent mutations in EGFR exons 19–23 detected in hepatocellular carcinoma tissues. [file 171845.f1.zip › 171845.f1/mat.171845.v2.pdf]

Supplement data

Table1. Association between EGFR overexpression and other variables in HCC tissues.

Variables	EGFR overexpression †		p^{*}
	Negative	Positive	
**Tissues**			
Matched non-cancerous (n=35)	25 (71.4%)	10 (28.6%)	
HCC (n=40)	27 (67.5%)	13 (32.5%)	0.713
**Age (Years)**			
<50	11 (57.9%)	8 (42.1%)	
≥50	16 (76.2%)	5 (23.8%)	0.217
**Sex**			
Male	22 (62.9%)	13 (37.1%)	
Female	5 (100%)	0 (0%)	0.154
**Tumor size**			
<5 cm	8 (61.5%)	5 (38.5%)	
≥5 cm	19 (70.4%)	8 (29.6%)	0.576
**AFP**			
<500 ng/ml	17 (70.8%)	7 (29.2%)	
≥500 ng/ml	8 (57.1%)	6 (42.9%)	0.391
**HBsAg**			
Negative	7 (63.6%)	4 (36.4%)	
Positive	20 (69%)	9 (36.4%)	0.748
**TP53 expression**			
Negative	14(70%)	6 (30%)	
Positive	13(65%)	7 (35%)	0.736
**Ki-67**			
Negative (<10%)	6 (66.7%)	3 (33.3%)	
Mildly positive (10-19%)	6 (75.0%)	2 (25.0%)	
Moderately positive (20-29%)	6 (54.5%)	5 (45.5%)	
Strongly positive (>30%)	9 (75.0%)	3 (25.0%)	0.894

† EGFR overexpression defined as negative (0 and 1+) and positive (2+ and 3+),* chi-square (χ^2) test

Table 2. Association between EGFR mutation and other variables in HCC tissues.

Variables	Missense mutation		*p**	Silent mutation		*p**
	Negative	Positive		Negative	Positive	
**Age (Years)**						
<50	10 (76.9%)	3 (23.1%)		6 (46.2%)	7 (53.8%)	
≥50	13 (65.0%)	7 (35.0%)	0.466	15 (75.0%)	5 (25.0%)	0.092
**Sex**						
Male	19 (70.4%)	8 (29.6%)		17 (63.0%)	10 (37%)	
Female	4 (66.7%)	2 (33.3%)	1.00	4 (66.7%)	2 (33.3%)	1.00
**Tumor size**						
<5 cm	10 (76.9%)	3 (23.1)		7 (53.8%)	6 (46.2%)	
≥5 cm	13 (65.0%)	7 (35.0%)	0.466	14 (70%)	6 (30%)	0.346
**AFP**						
<500 ng/ml	15 (68.2%)	7 (31.8%)		13 (59.1%)	9 (40.9%)	
≥500 ng/ml	7 (70.0%)	3 (30.0%)	0.918	7 (70%)	3 (30%)	0.555
**HBsAg**						
Negative	16 (76.2%)	5 (23.8%)		13 (61.9%)	8 (38.1%)	
Positive	7 (63.6%)	4 (36.4%)	0.453	7 (63.6%)	4 (36.4%)	0.923
**TP53 expression**						
Negative	12 (70.6%)	5 (29.4%)		8 (47.1%)	9 (52.9%)	
Positive	8 (66.7%)	4 (33.3%)	0.822	9 (75.0%)	3 (25.0%)	0.132
**Ki-67**						
Negative (<10%)	5 (62.5%)	3 (37.5%)		4 (50.0%)	4 (50.0%)	
Mildly positive (10-19%)	4 (80.0%)	1 (20.0%)		4(80.0%)	1 (20.0%)	
Moderately positive (20-29%)	6 (85.7%)	1 (14.3%)		4(57.1%)	3 (42.9%)	
Strongly positive (>30%)	5 (55.6%)	4 (44.4%)	0.810	5(55.6%)	4 (44.4%)	0.991
**EGFR overexpression**						
Negative (score 0, 1+)	13 (68.4%)	6 (31.6%)		13(68.4%)	6 (31.6%)	
Positive (score 2+, 3+)	6 (66.7%)	3 (33.3%)	0.926	3(33.3%)	6 (66.7%)	0.080

* chi-square (χ^2) test
