# Supplementary material for: Missense Mutations in Exons 18–24 of EGFR in Hepatocellular Carcinoma Tissues
Source: Biomed Res Int. 2015 Sep 7;2015:171845. doi: 10.1155/2015/171845 (PMC4575985; doi:10.1155/2015/171845)
Supplement: Supplementary file 1 — Table 1. Association between EGFR overexpression and other variables in HCC tissues. Table 2. Association between EGFR mutation and other variables in HCC tissues. Electropherograms of the 13 missense mutations in EGFR exons 19–23 detected in hepatocellular carcinoma tissues. Electropherograms of the 11 silent mutations in EGFR exons 19–23 detected in hepatocellular carcinoma tissues. [file 171845.f1.zip › 171845.f1/Missence mutations in HCC for supplement data BMRI.pptx]

## Slide 1
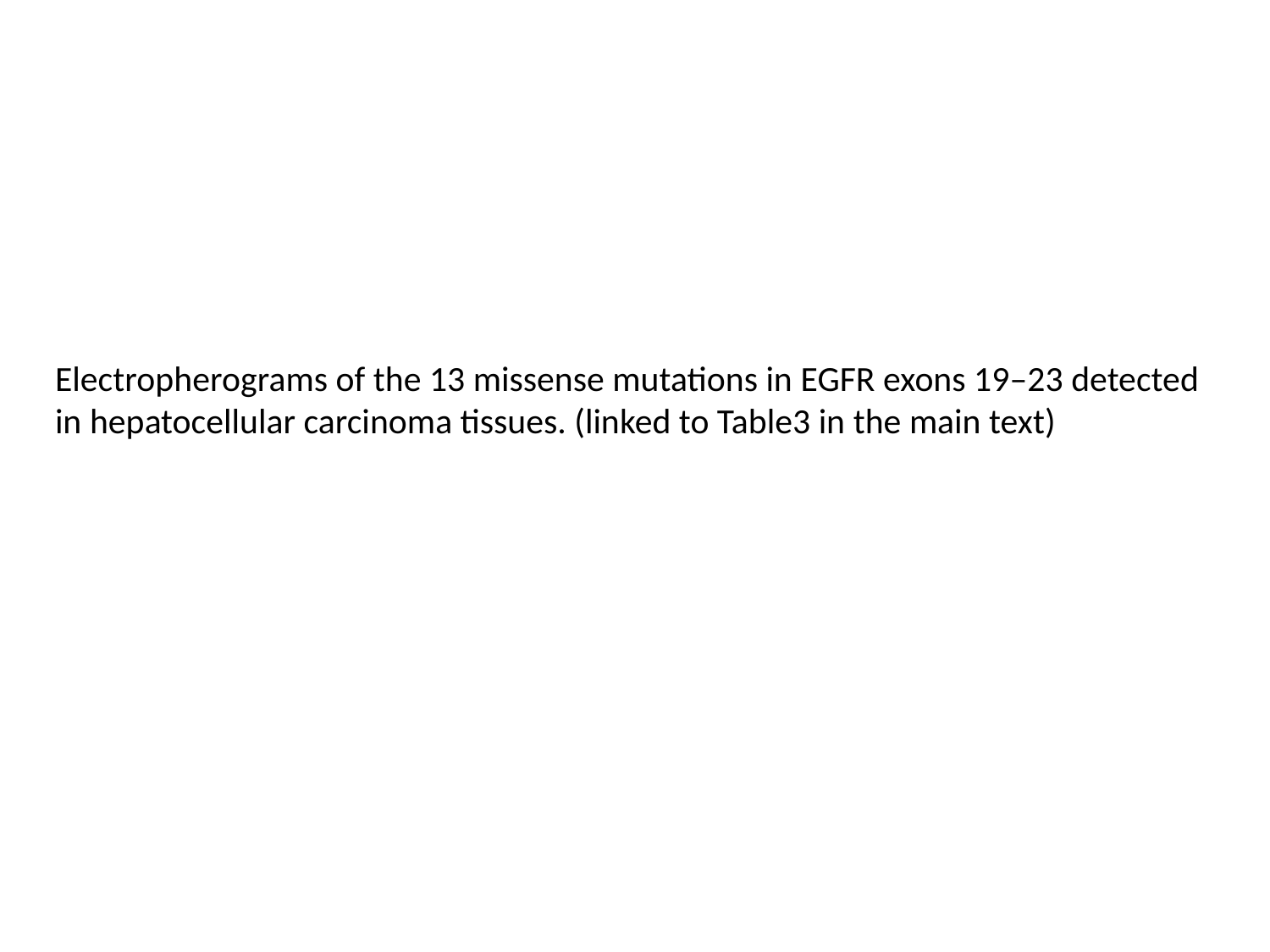

# Electropherograms of the 13 missense mutations in EGFR exons 19–23 detected in hepatocellular carcinoma tissues. (linked to Table3 in the main text)

## Slide 2
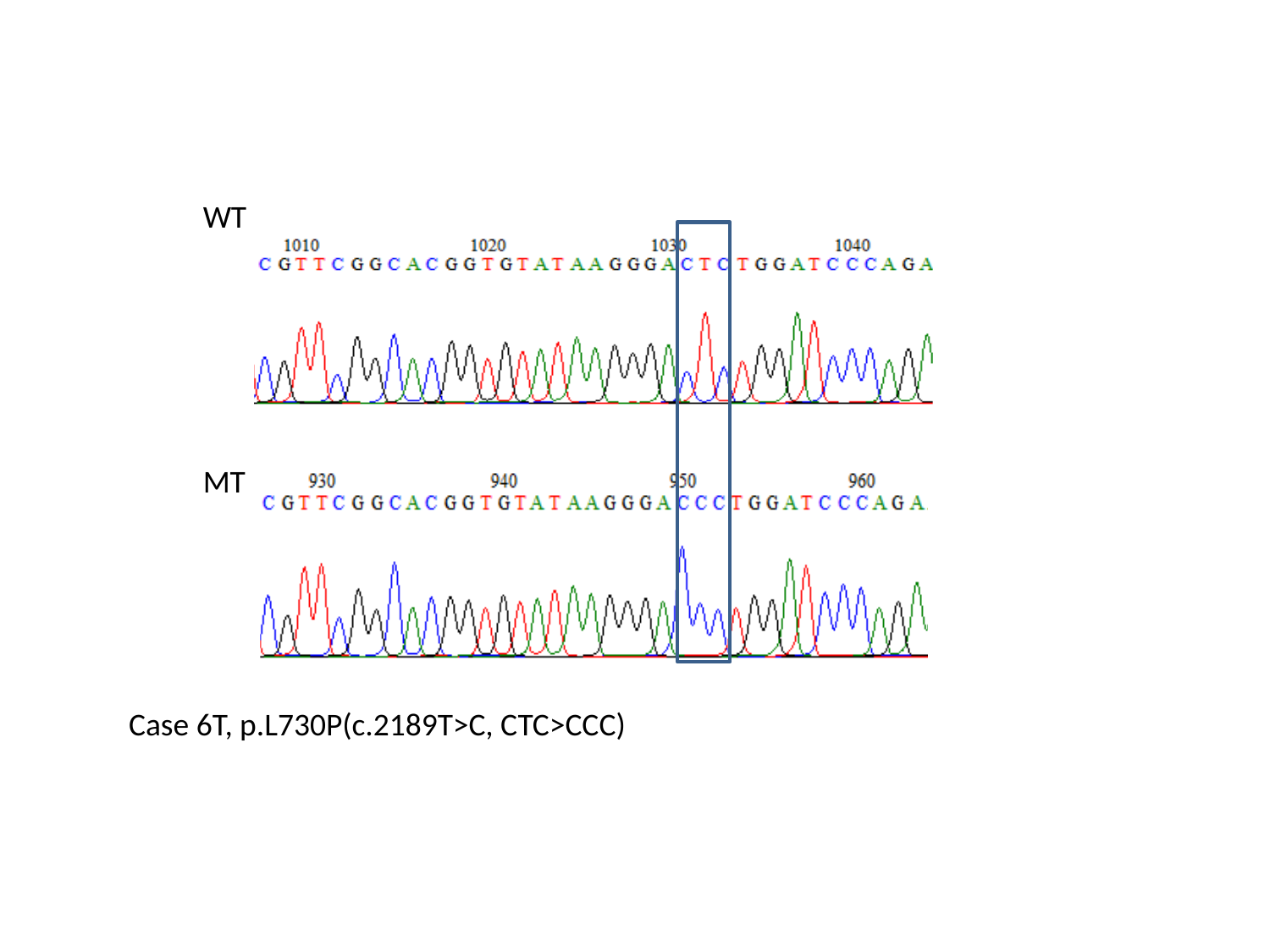

WT
MT
Case 6T, p.L730P(c.2189T>C, CTC>CCC)

## Slide 3
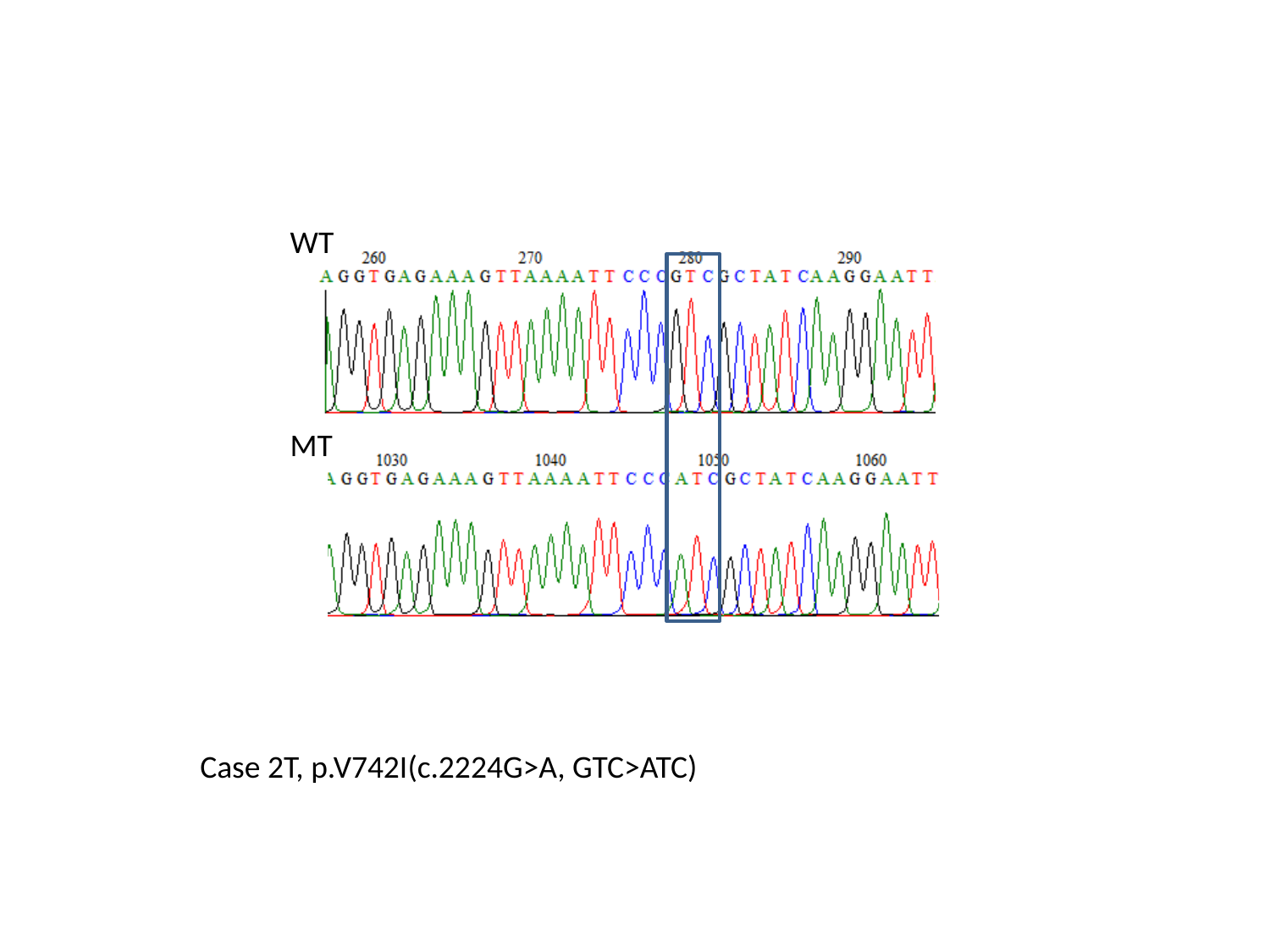

WT
MT
Case 2T, p.V742I(c.2224G>A, GTC>ATC)

## Slide 4
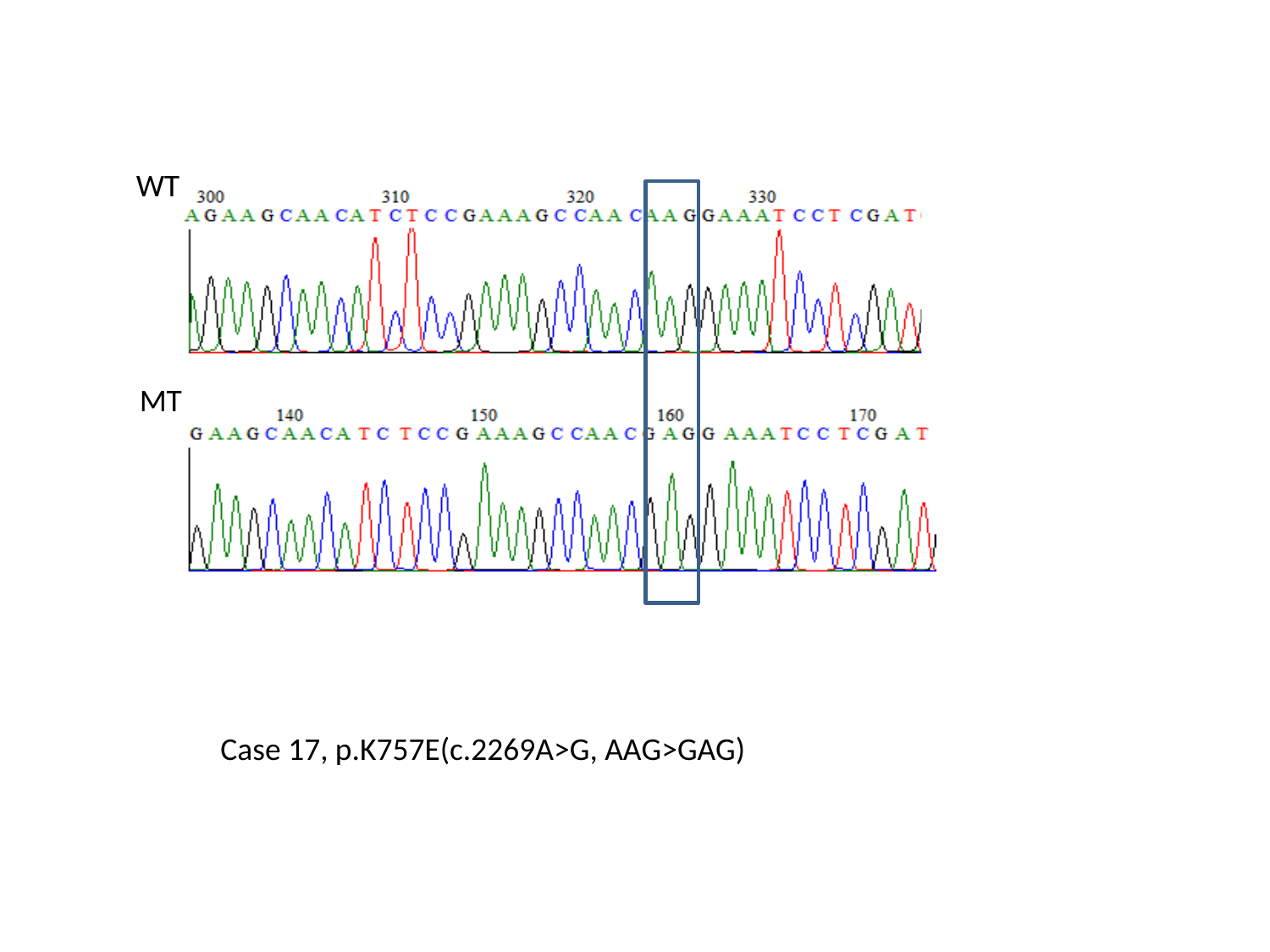

WT
MT
Case 17, p.K757E(c.2269A>G, AAG>GAG)

## Slide 5
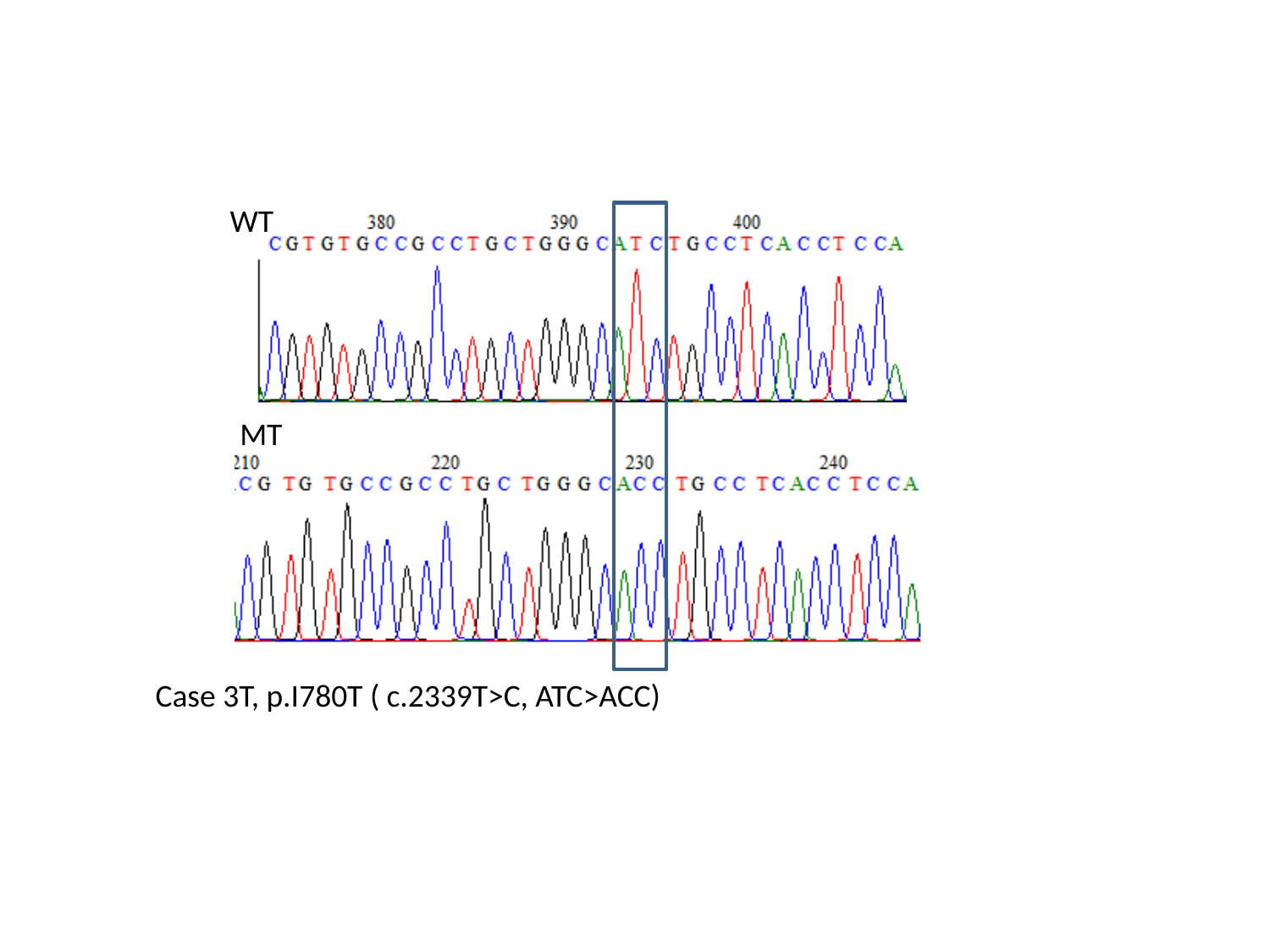

WT
MT
Case 3T, p.I780T ( c.2339T>C, ATC>ACC)

## Slide 6
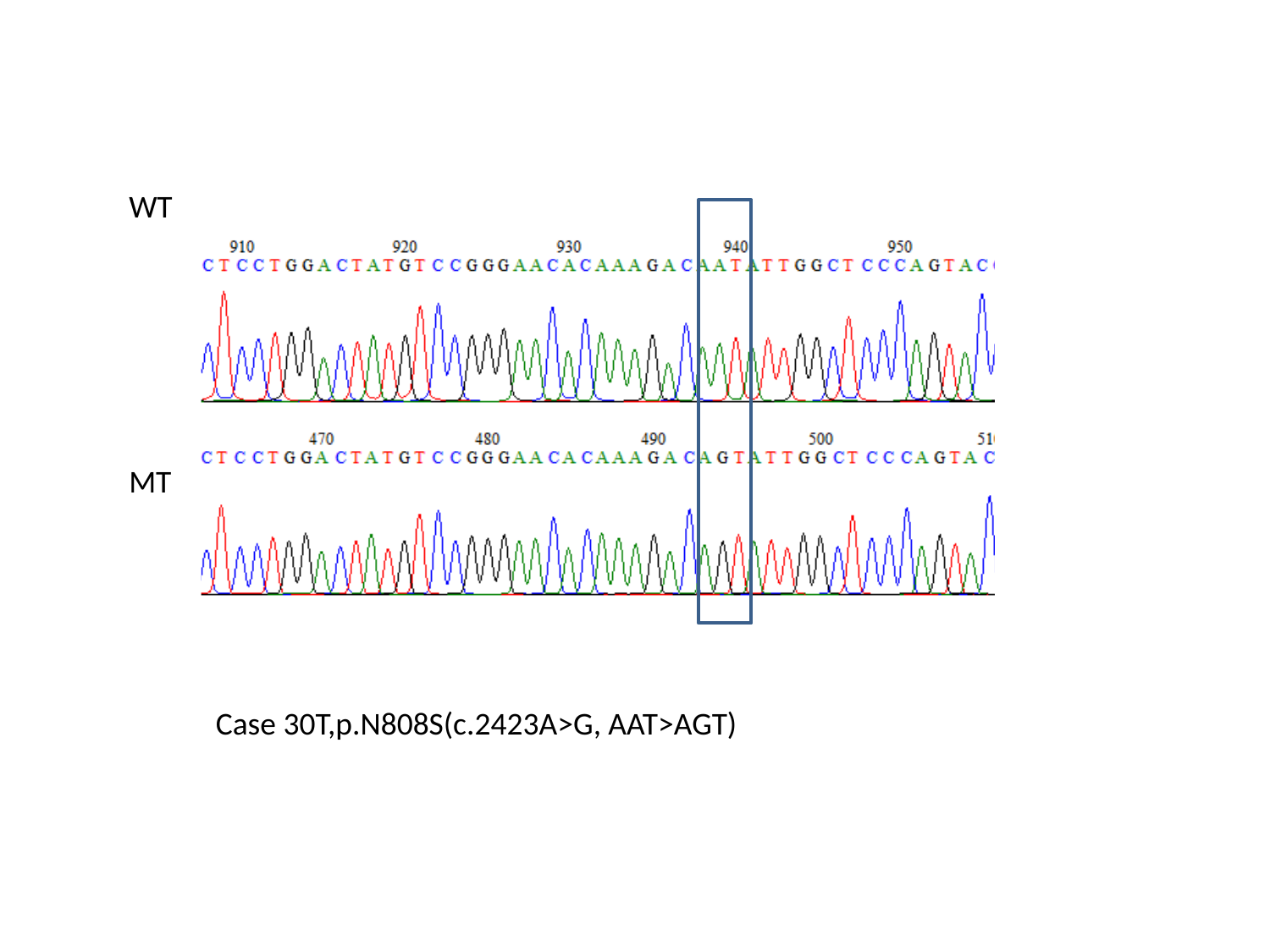

WT
MT
Case 30T,p.N808S(c.2423A>G, AAT>AGT)

## Slide 7
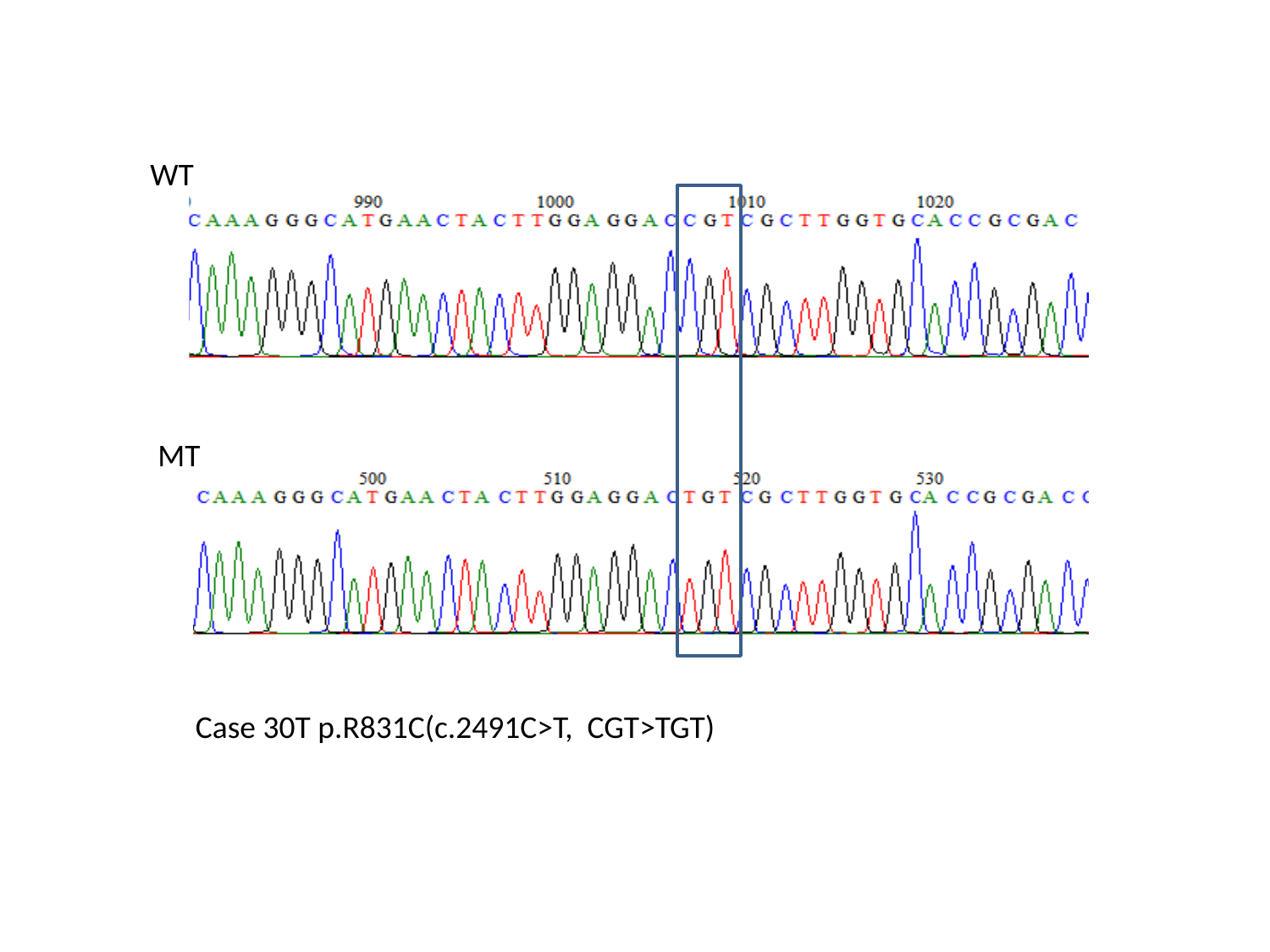

WT
MT
Case 30T p.R831C(c.2491C>T, CGT>TGT)

## Slide 8
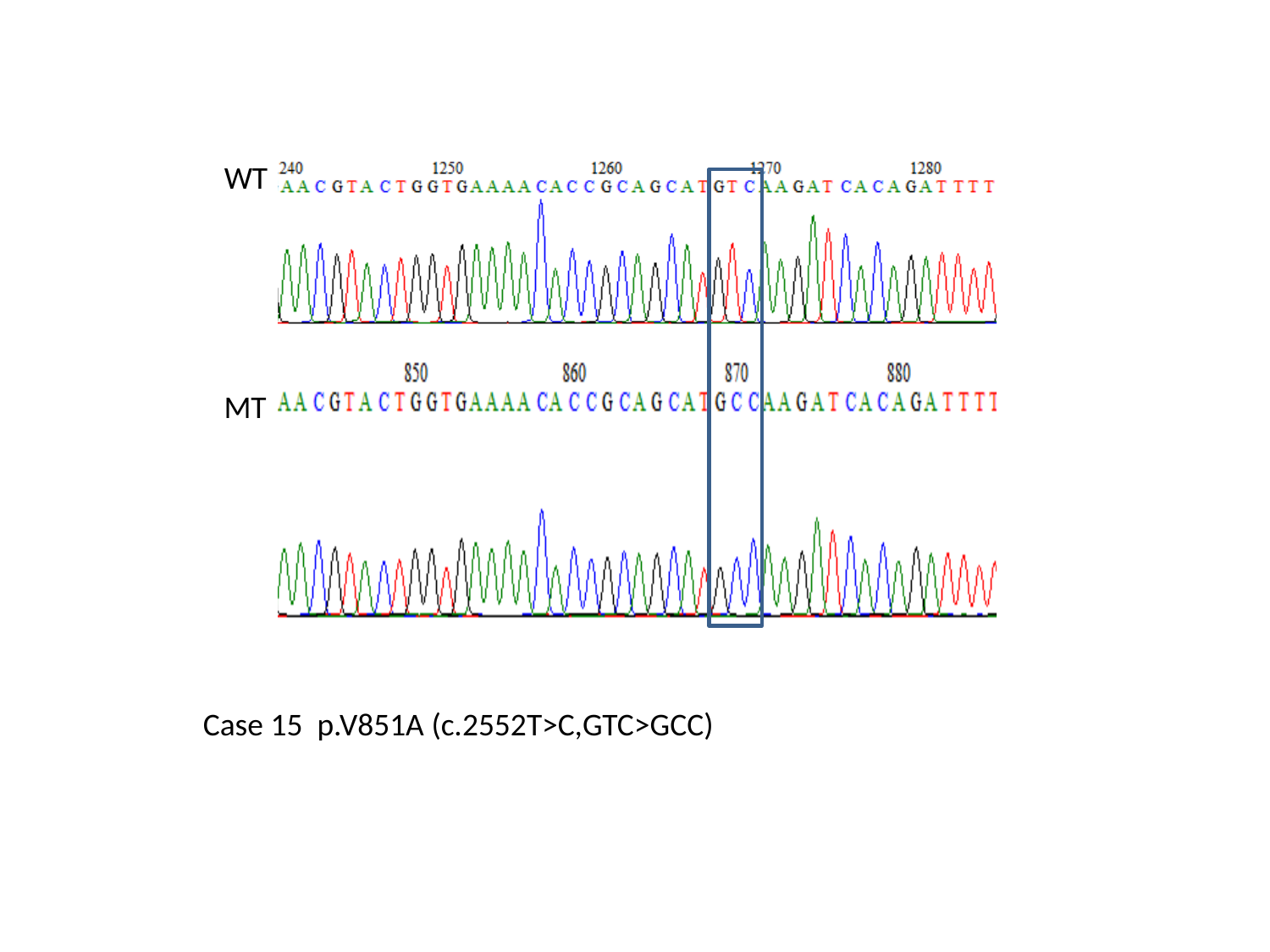

WT
MT
Case 15 p.V851A (c.2552T>C,GTC>GCC)

## Slide 9
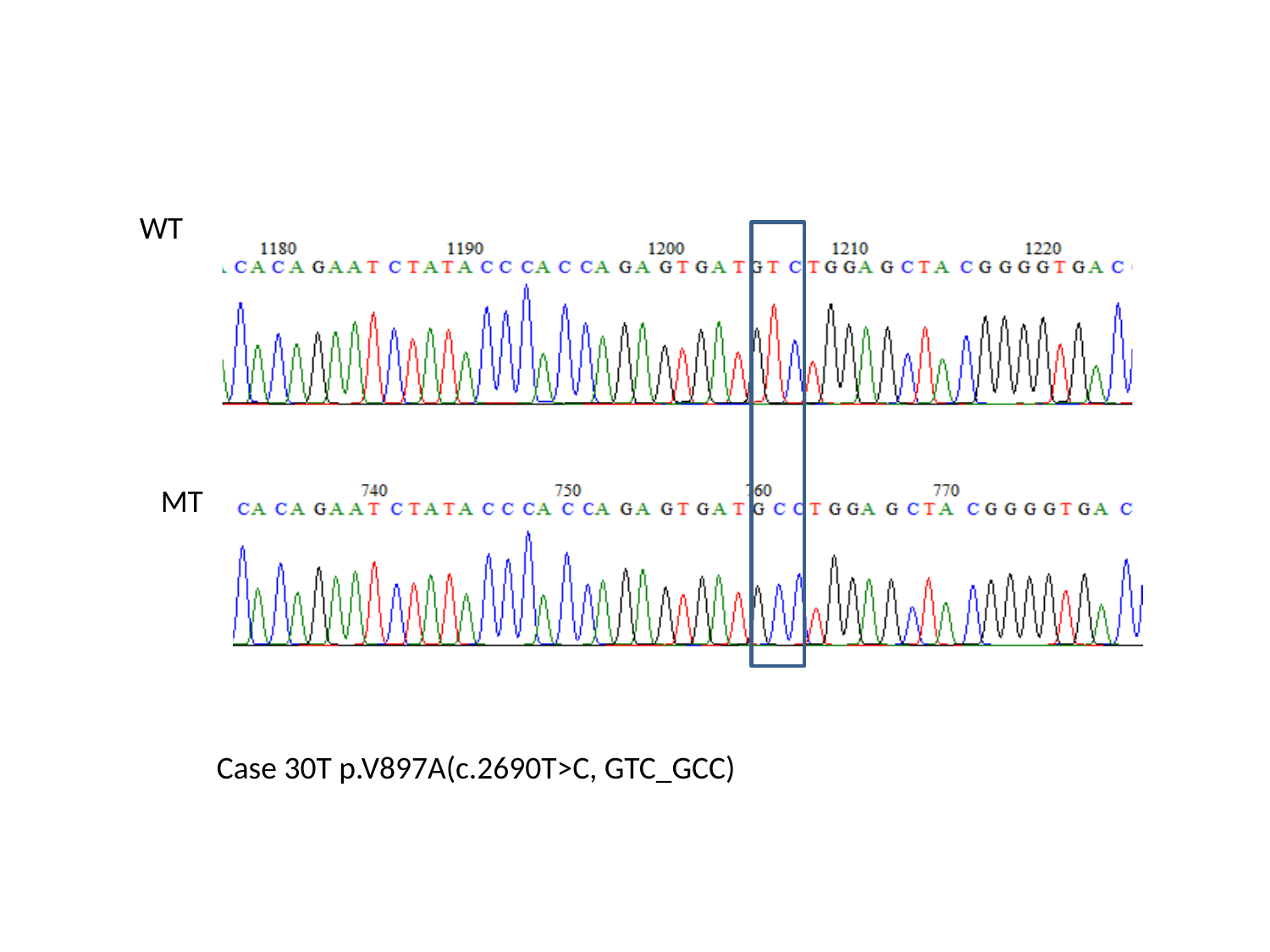

WT
MT
Case 30T p.V897A(c.2690T>C, GTC_GCC)

## Slide 10
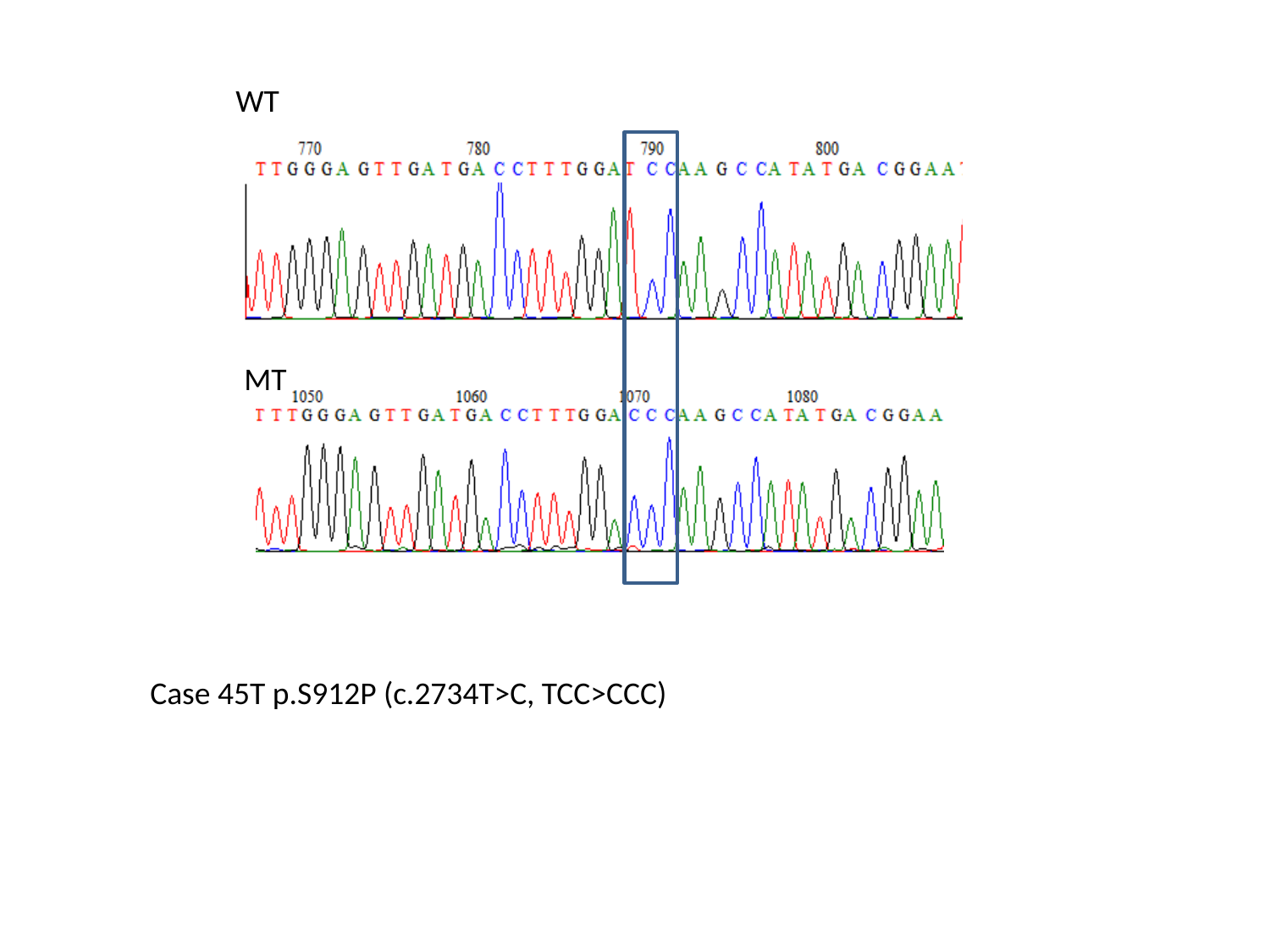

WT
MT
Case 45T p.S912P (c.2734T>C, TCC>CCC)

## Slide 11
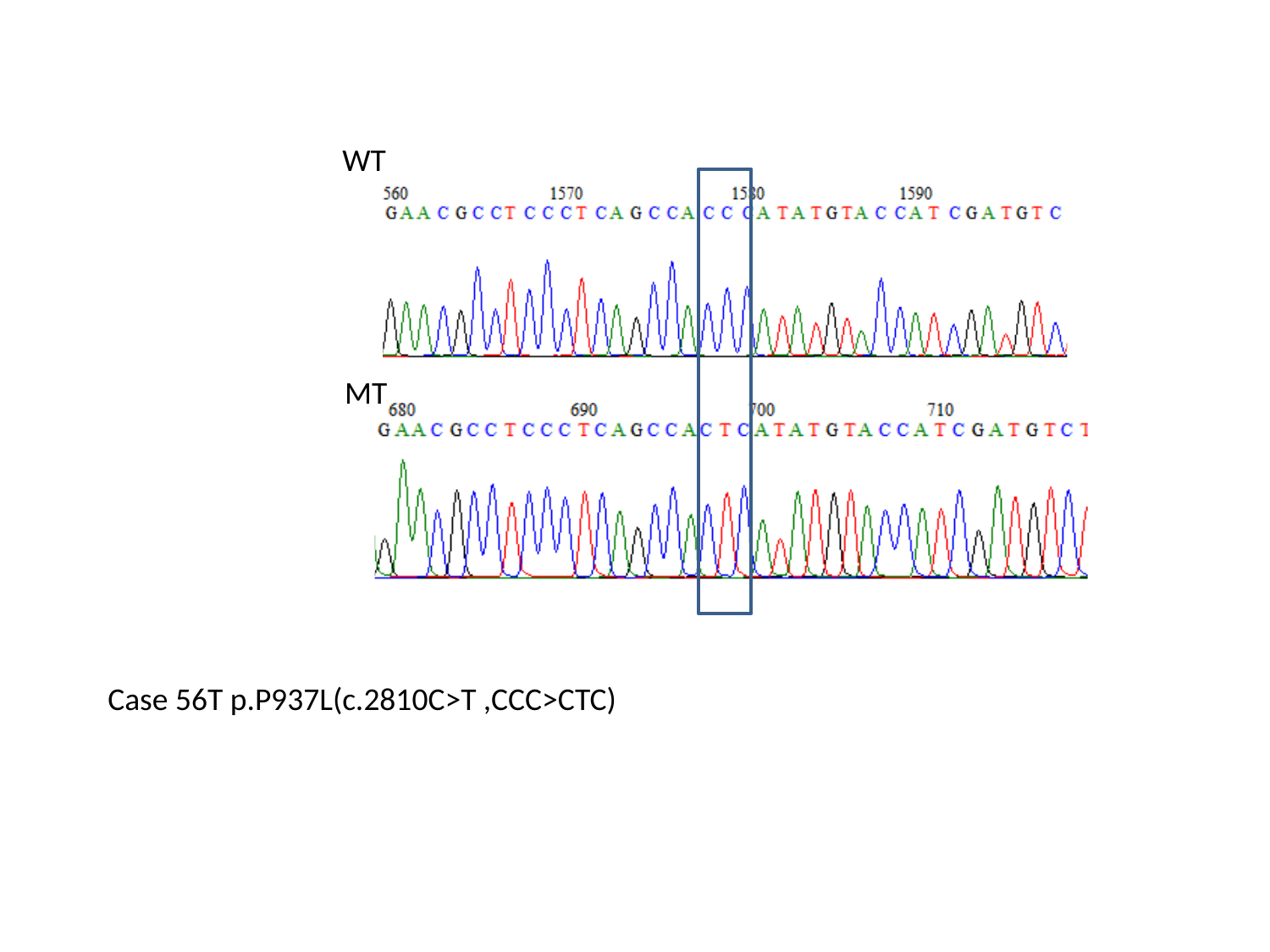

WT
MT
Case 56T p.P937L(c.2810C>T ,CCC>CTC)

## Slide 12
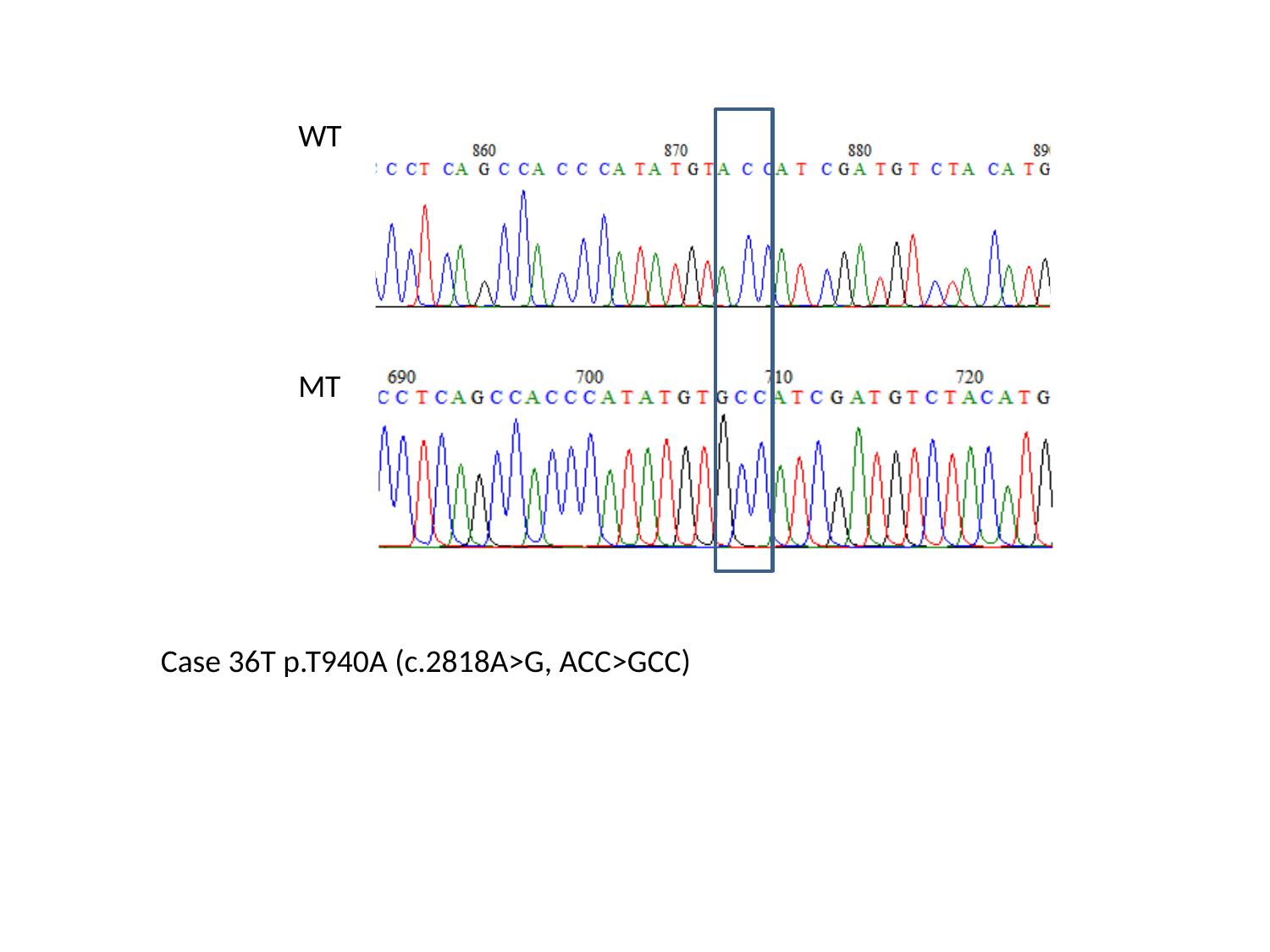

WT
MT
Case 36T p.T940A (c.2818A>G, ACC>GCC)

## Slide 13
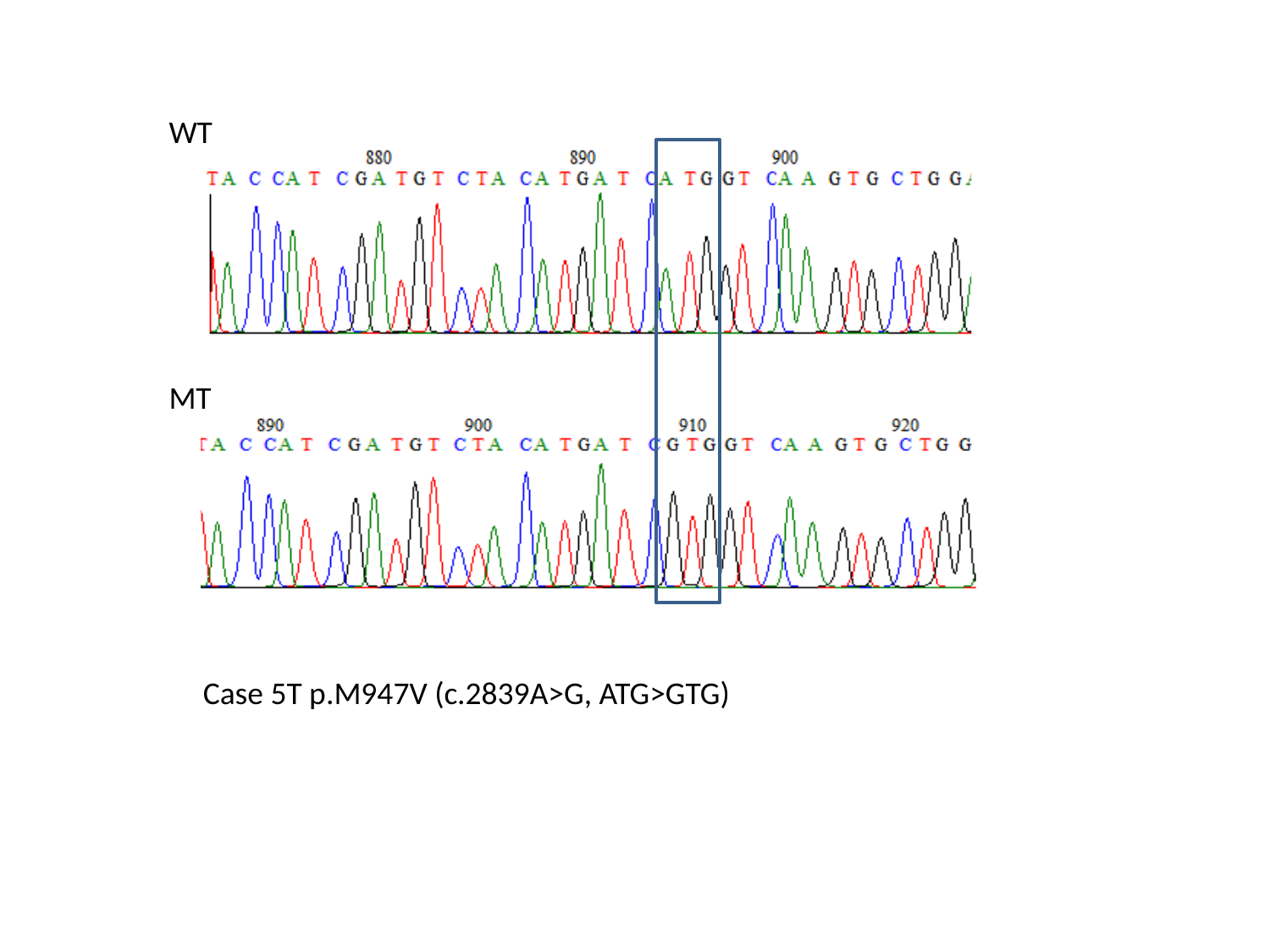

WT
MT
Case 5T p.M947V (c.2839A>G, ATG>GTG)

## Slide 14
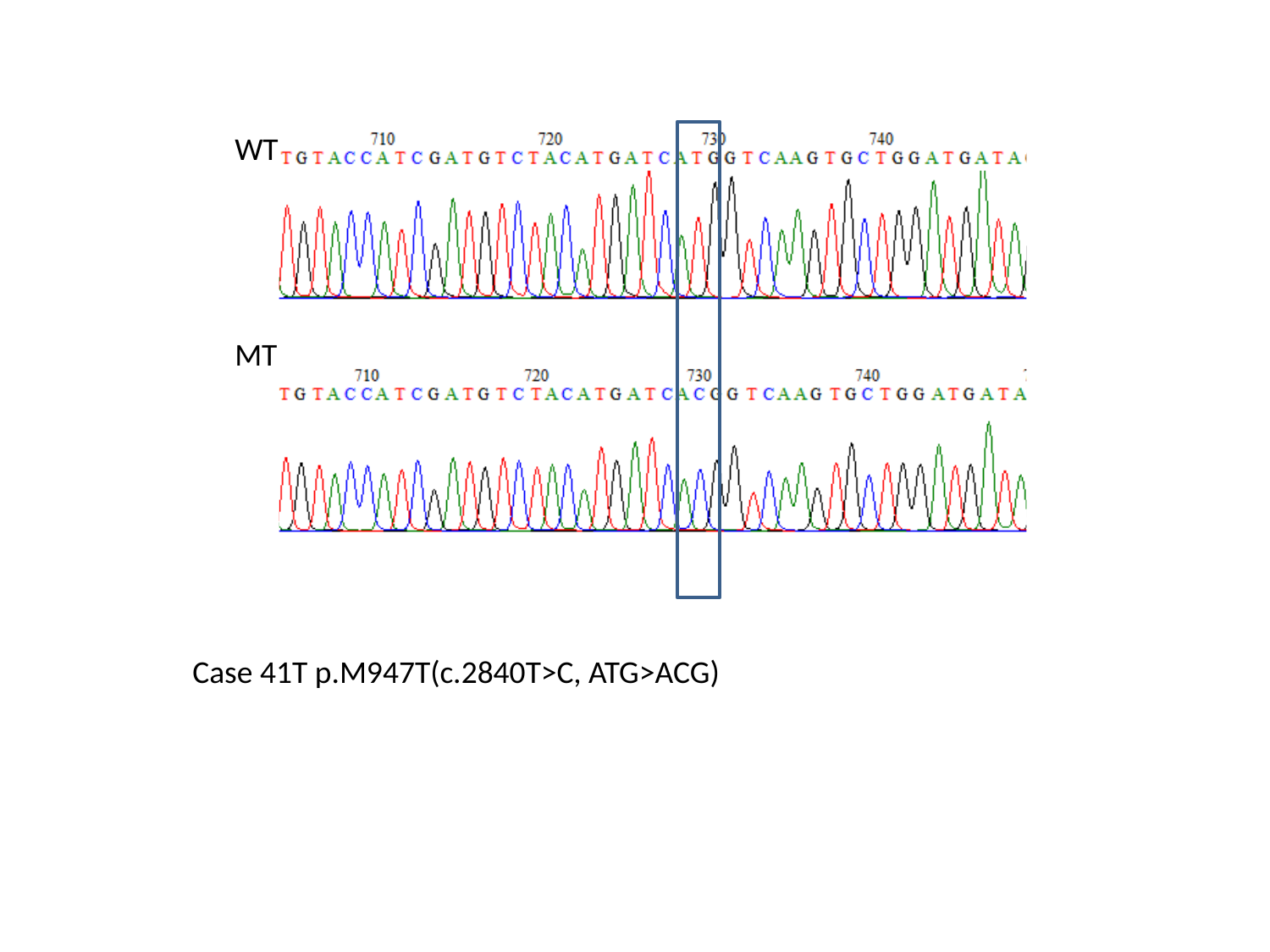

WT
MT
Case 41T p.M947T(c.2840T>C, ATG>ACG)
