# Supplementary material for: Missense Mutations in Exons 18–24 of EGFR in Hepatocellular Carcinoma Tissues
Source: Biomed Res Int. 2015 Sep 7;2015:171845. doi: 10.1155/2015/171845 (PMC4575985; doi:10.1155/2015/171845)
Supplement: Supplementary file 1 — Table 1. Association between EGFR overexpression and other variables in HCC tissues. Table 2. Association between EGFR mutation and other variables in HCC tissues. Electropherograms of the 13 missense mutations in EGFR exons 19–23 detected in hepatocellular carcinoma tissues. Electropherograms of the 11 silent mutations in EGFR exons 19–23 detected in hepatocellular carcinoma tissues. [file 171845.f1.zip › 171845.f1/Silent mutations in HCC for supplement data BMRI.pptx]

## Slide 1
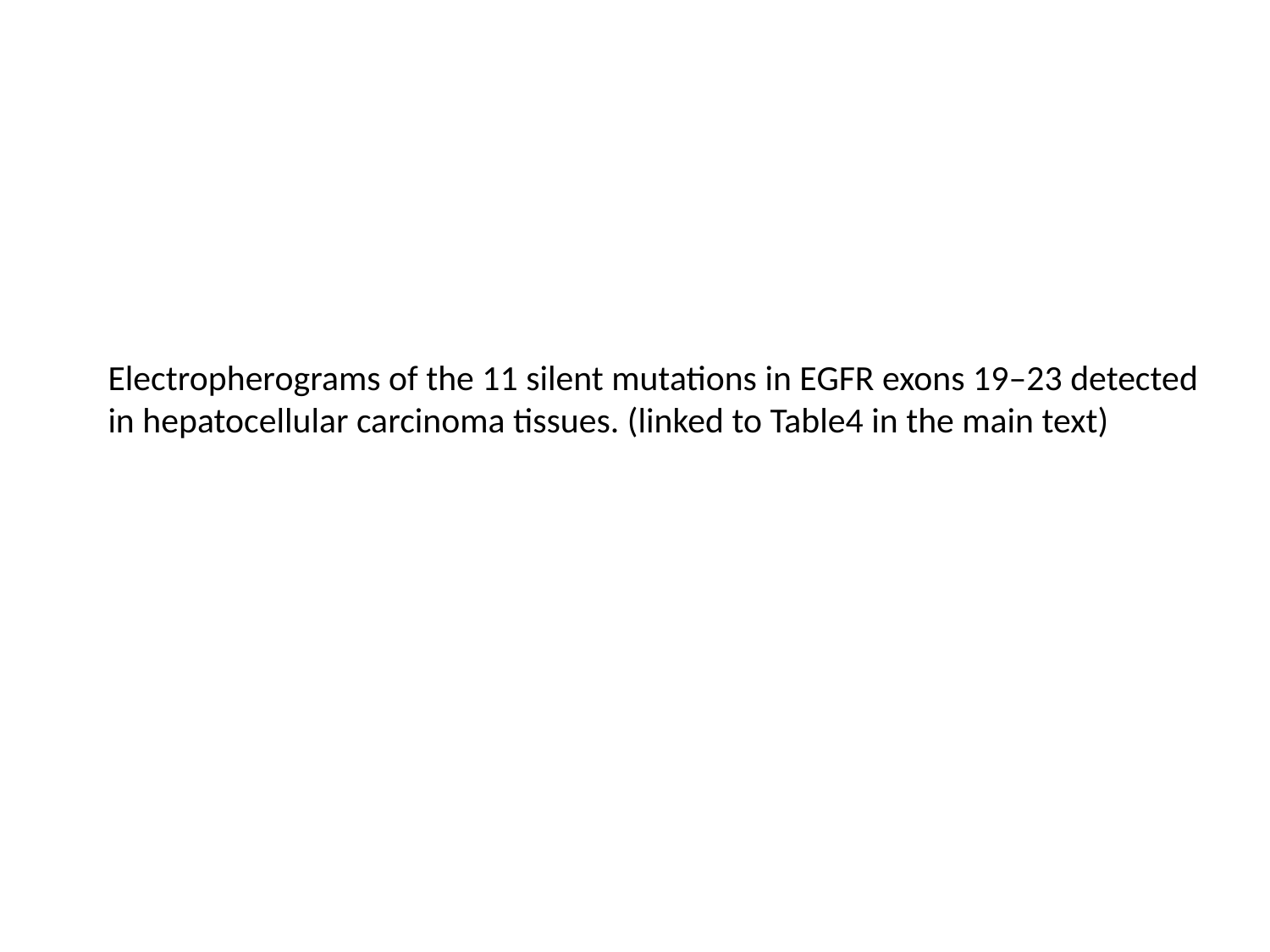

# Electropherograms of the 11 silent mutations in EGFR exons 19–23 detected in hepatocellular carcinoma tissues. (linked to Table4 in the main text)

## Slide 2
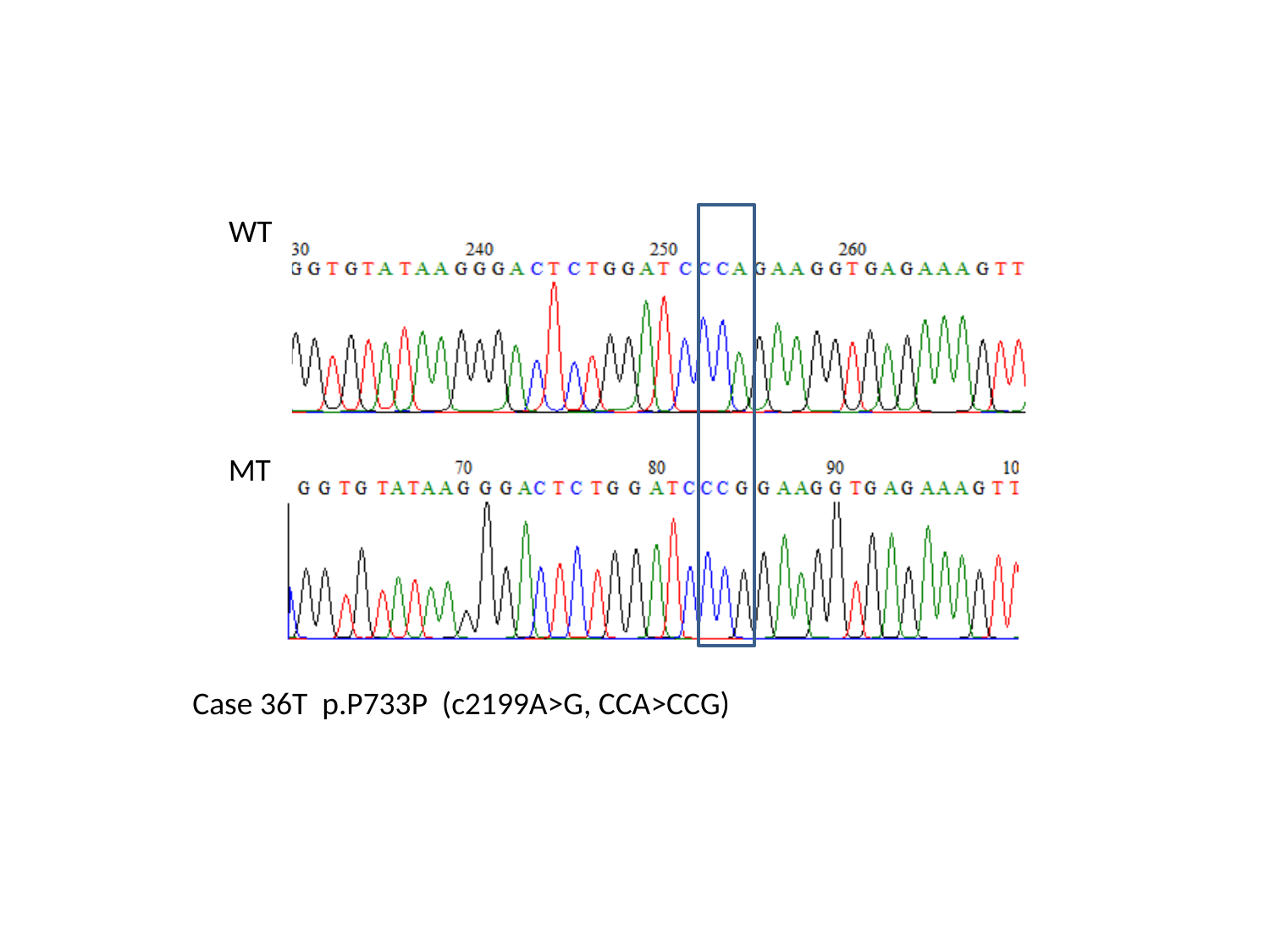

WT
MT
Case 36T p.P733P (c2199A>G, CCA>CCG)

## Slide 3
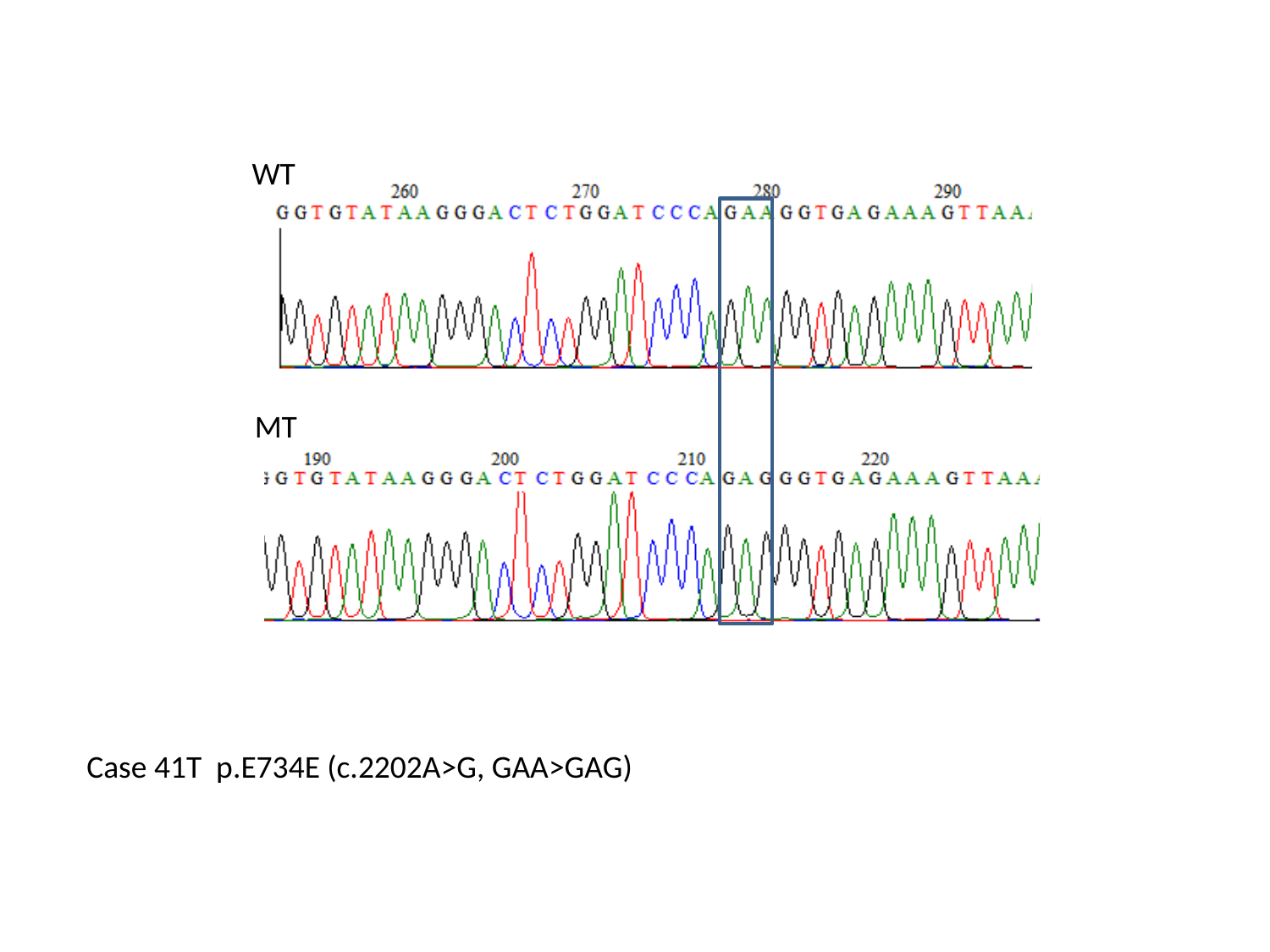

WT
MT
Case 41T p.E734E (c.2202A>G, GAA>GAG)

## Slide 4
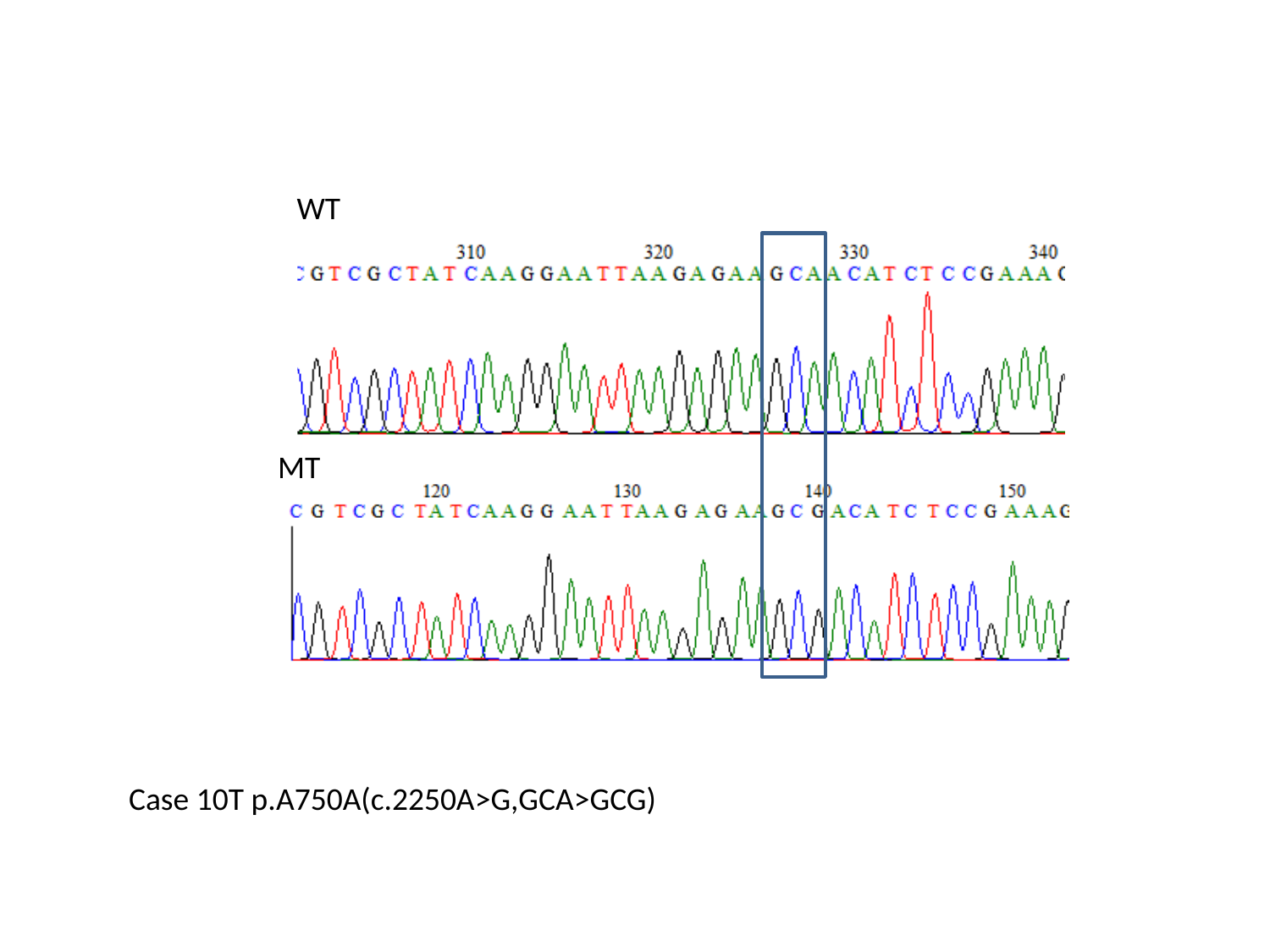

WT
MT
Case 10T p.A750A(c.2250A>G,GCA>GCG)

## Slide 5
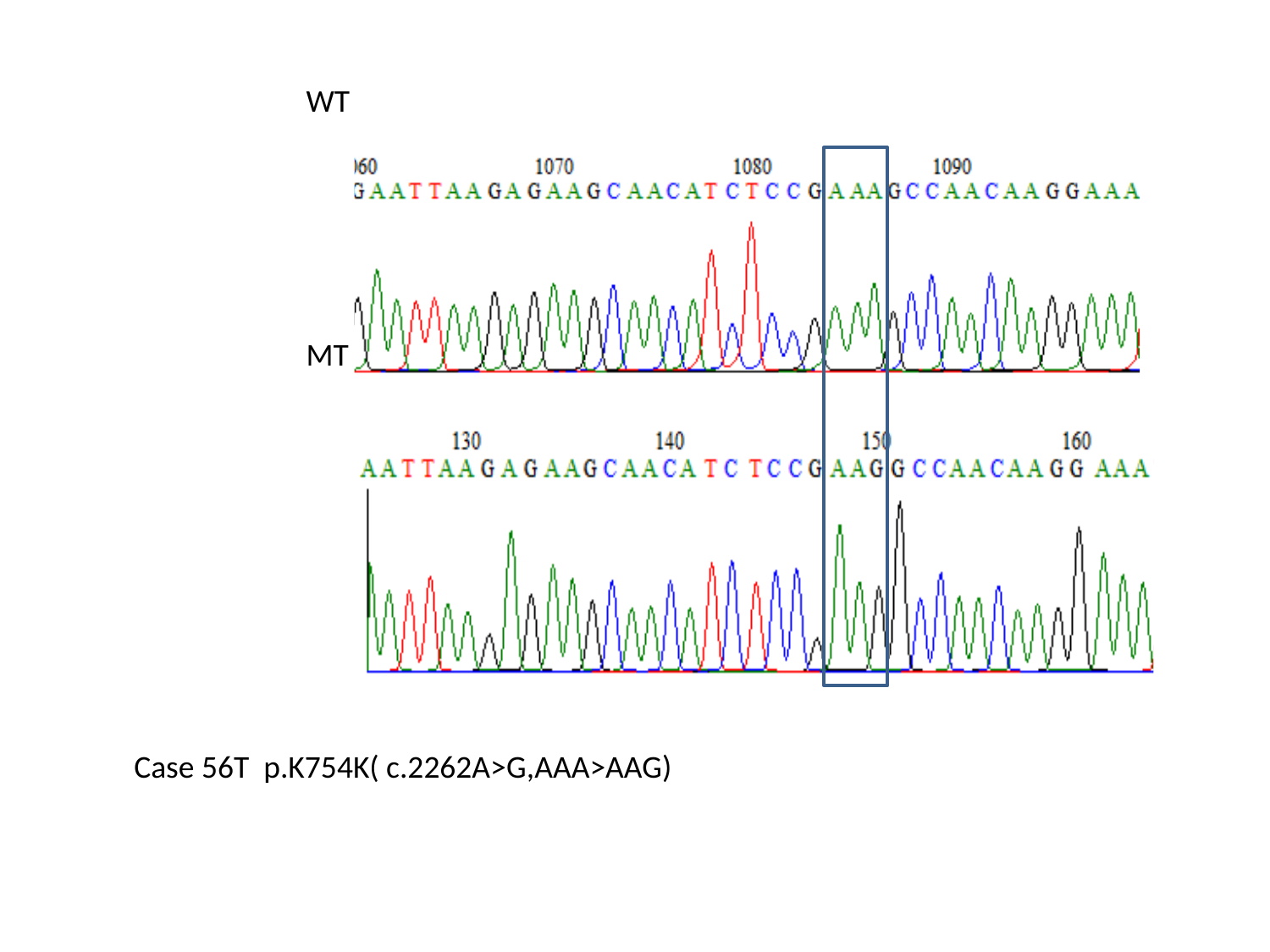

WT
MT
Case 56T p.K754K( c.2262A>G,AAA>AAG)

## Slide 6
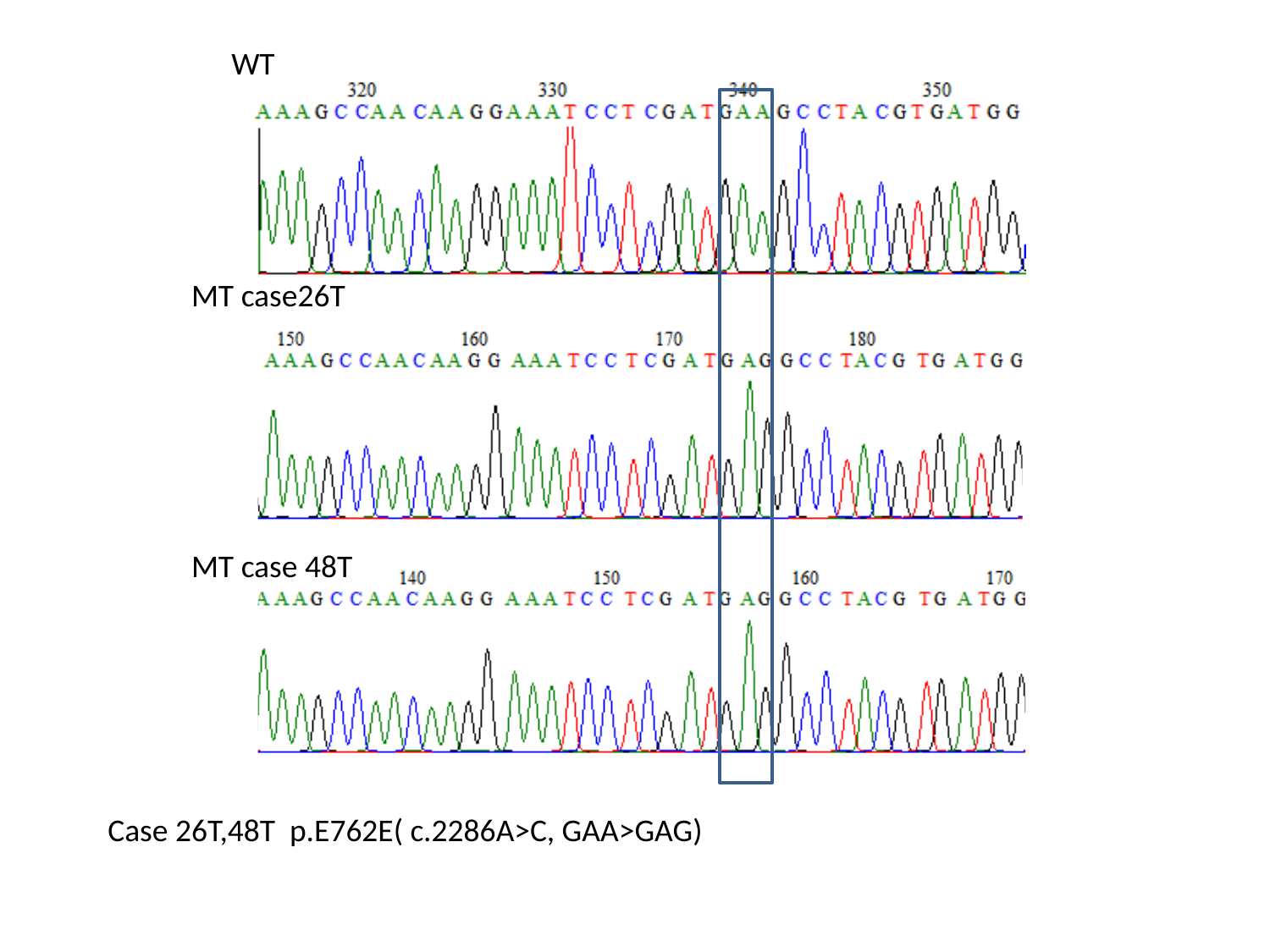

WT
MT case26T
MT case 48T
Case 26T,48T p.E762E( c.2286A>C, GAA>GAG)

## Slide 7
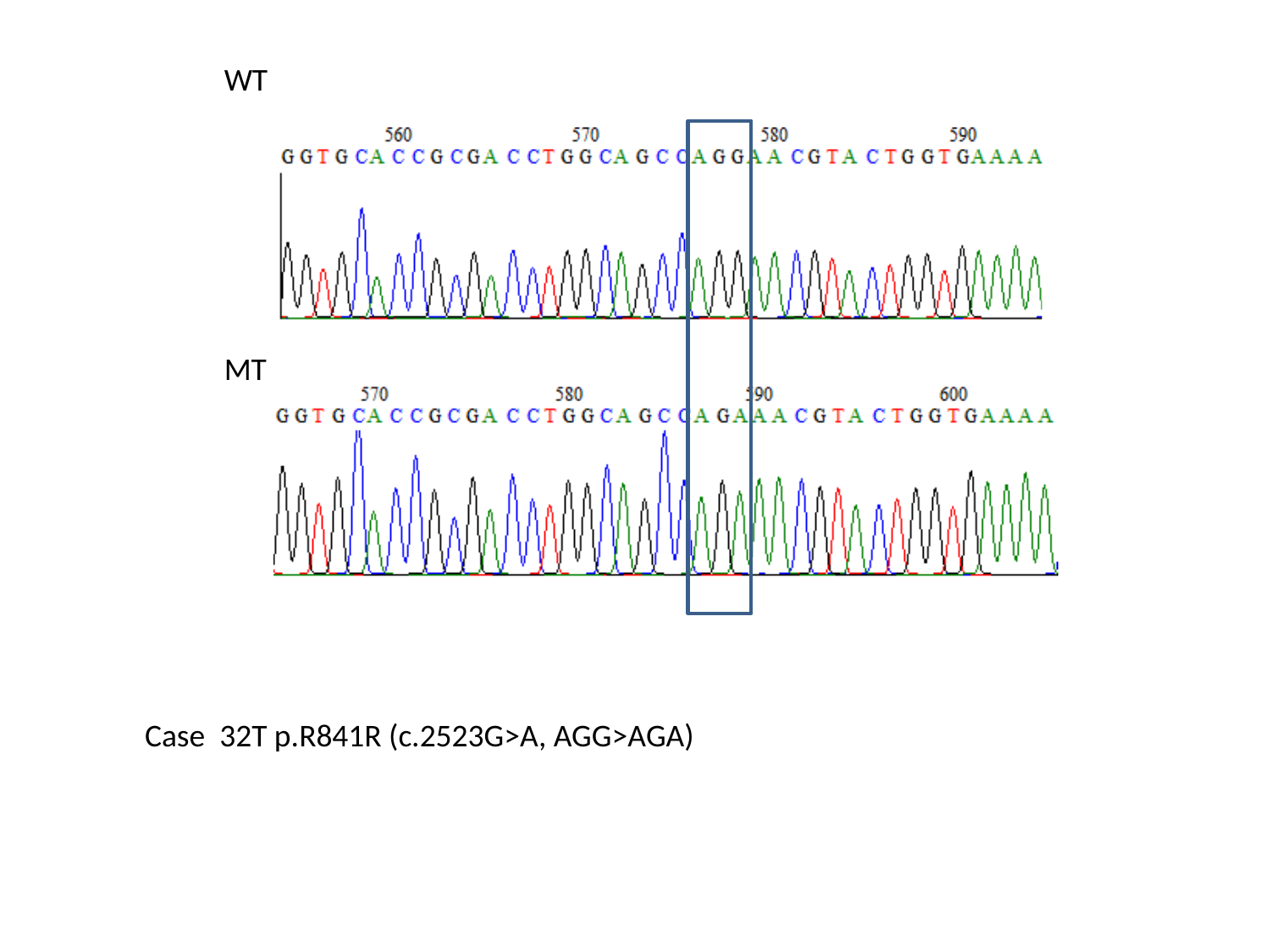

WT
MT
Case 32T p.R841R (c.2523G>A, AGG>AGA)

## Slide 8
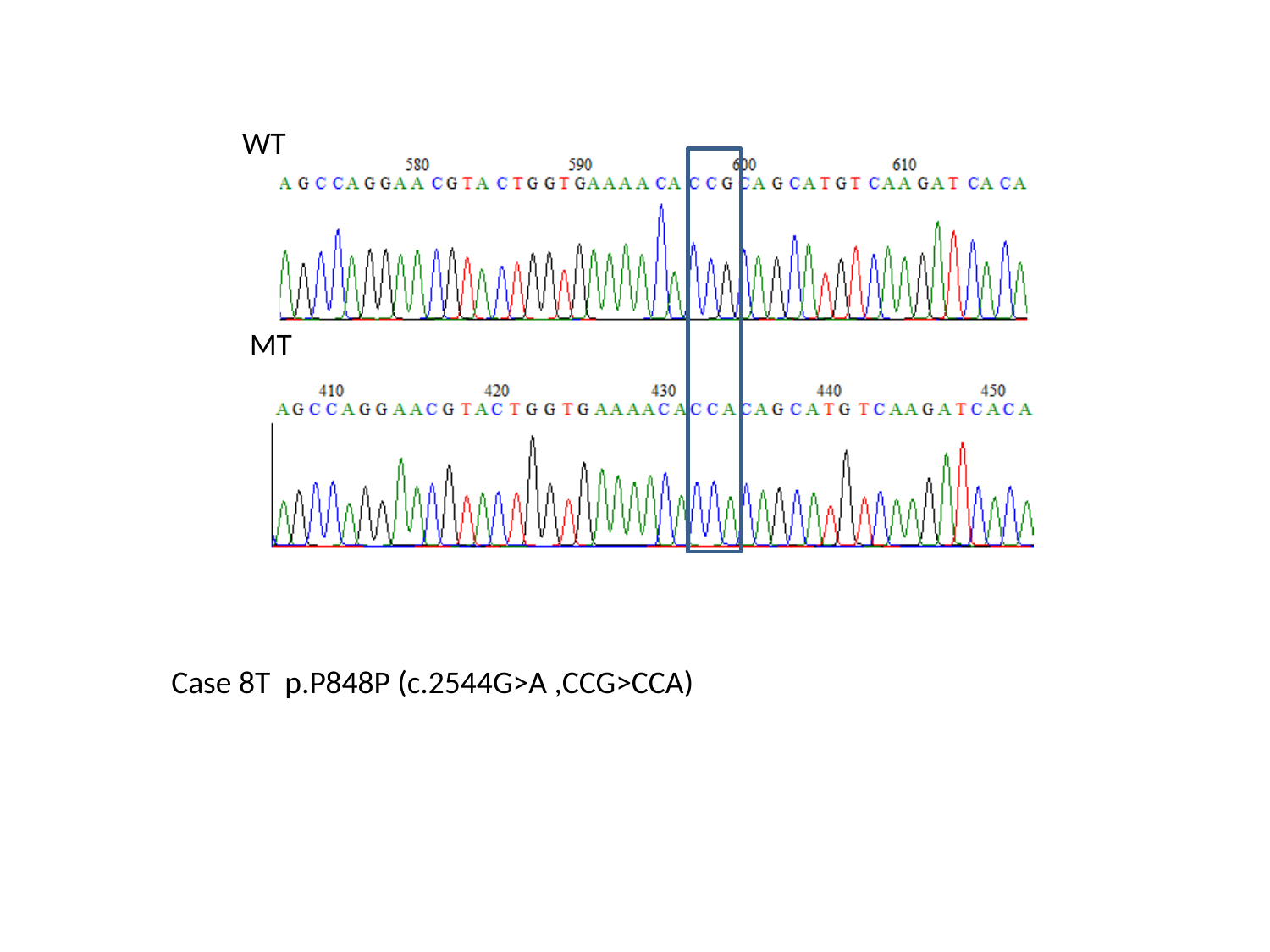

WT
MT
Case 8T p.P848P (c.2544G>A ,CCG>CCA)

## Slide 9
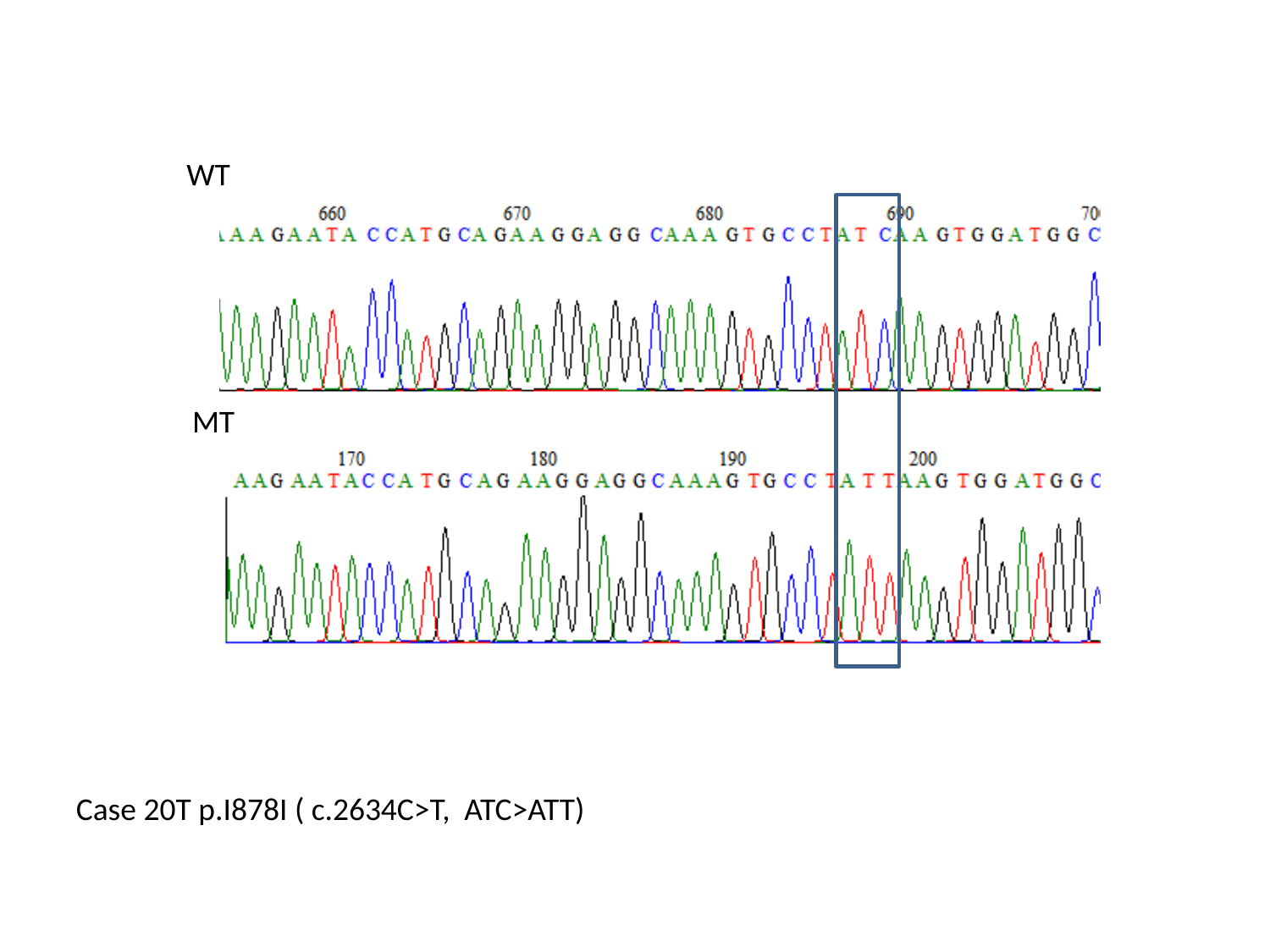

WT
MT
Case 20T p.I878I ( c.2634C>T, ATC>ATT)

## Slide 10
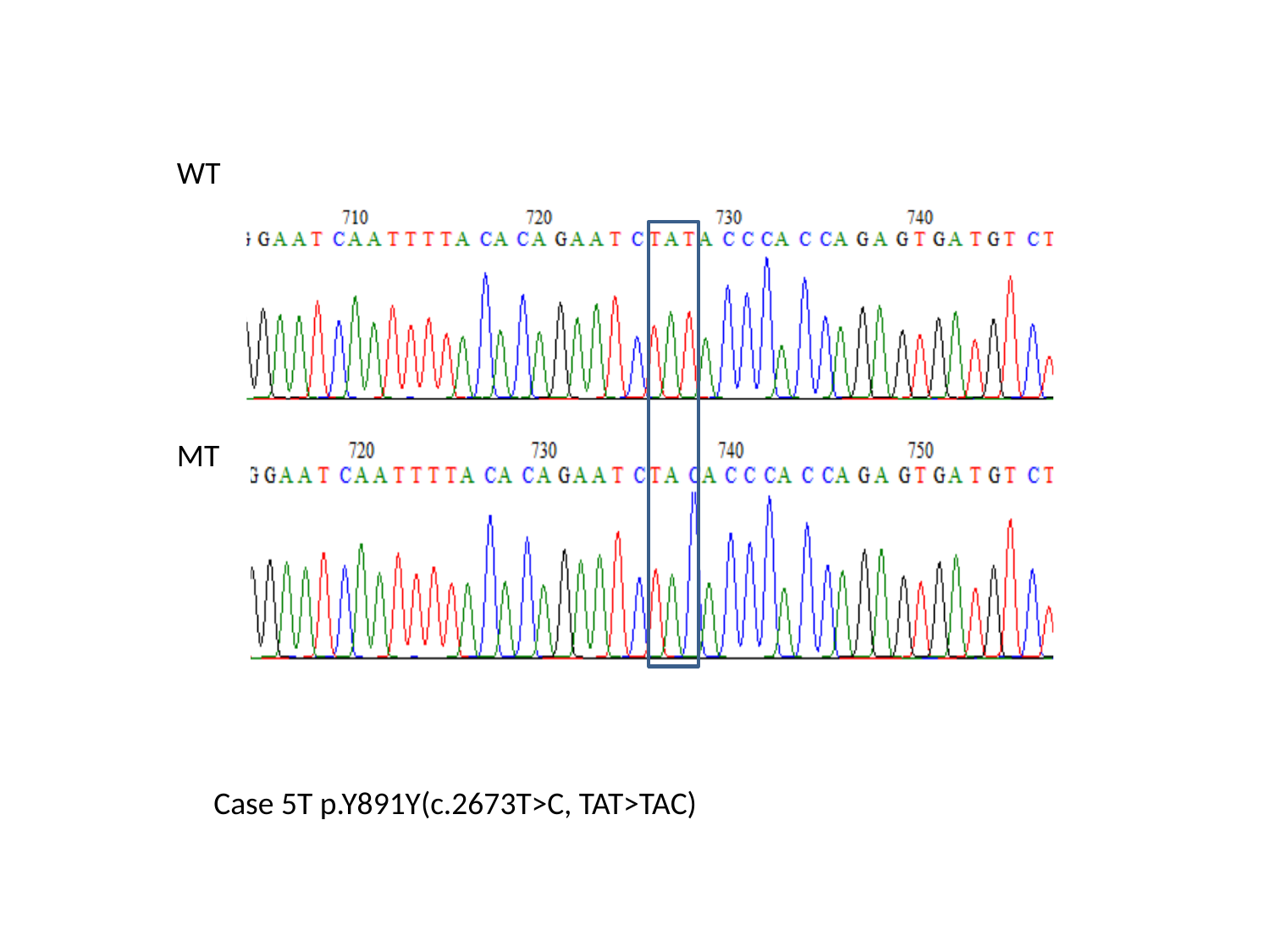

WT
MT
Case 5T p.Y891Y(c.2673T>C, TAT>TAC)

## Slide 11
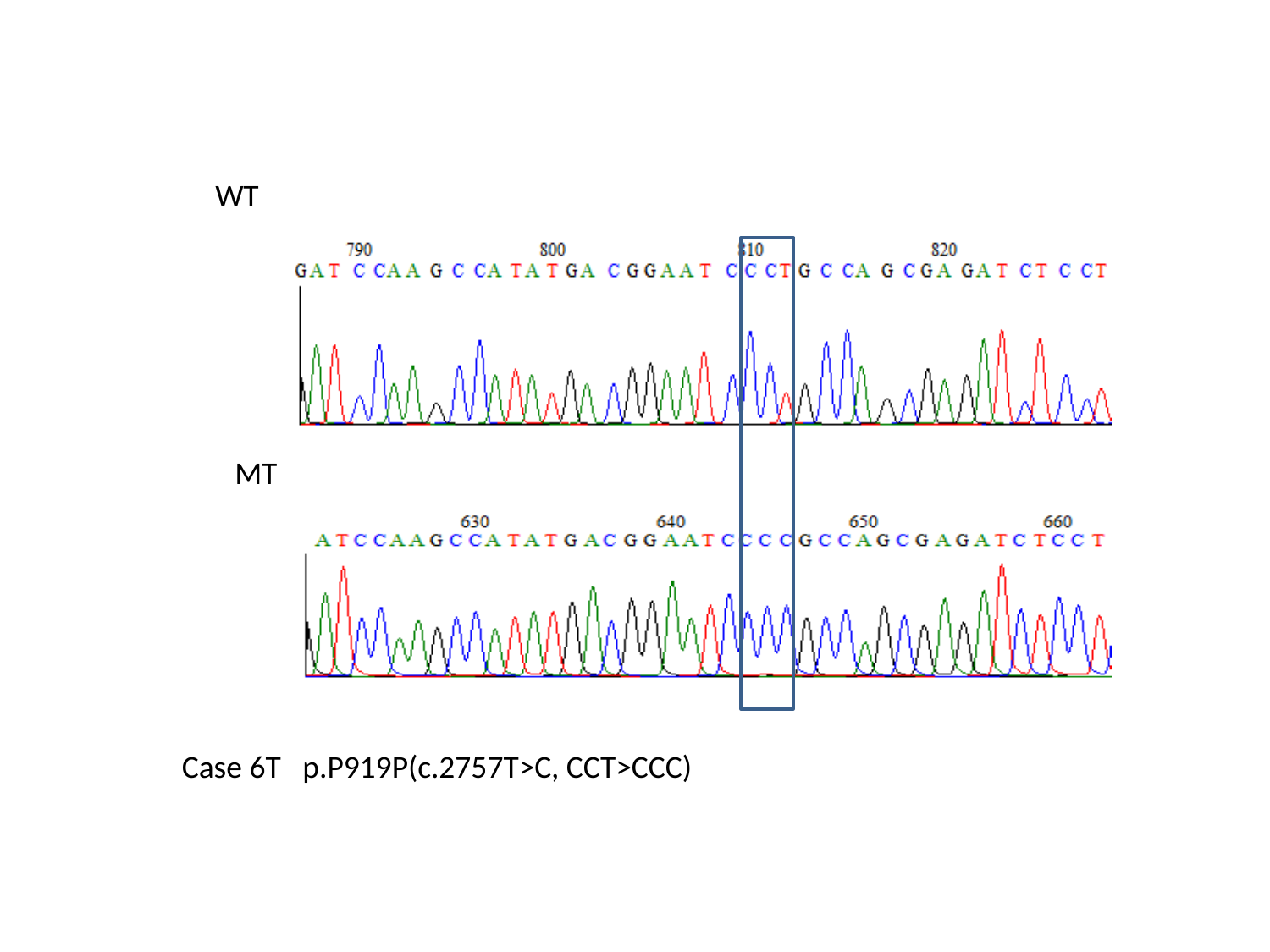

WT
MT
Case 6T p.P919P(c.2757T>C, CCT>CCC)

## Slide 12
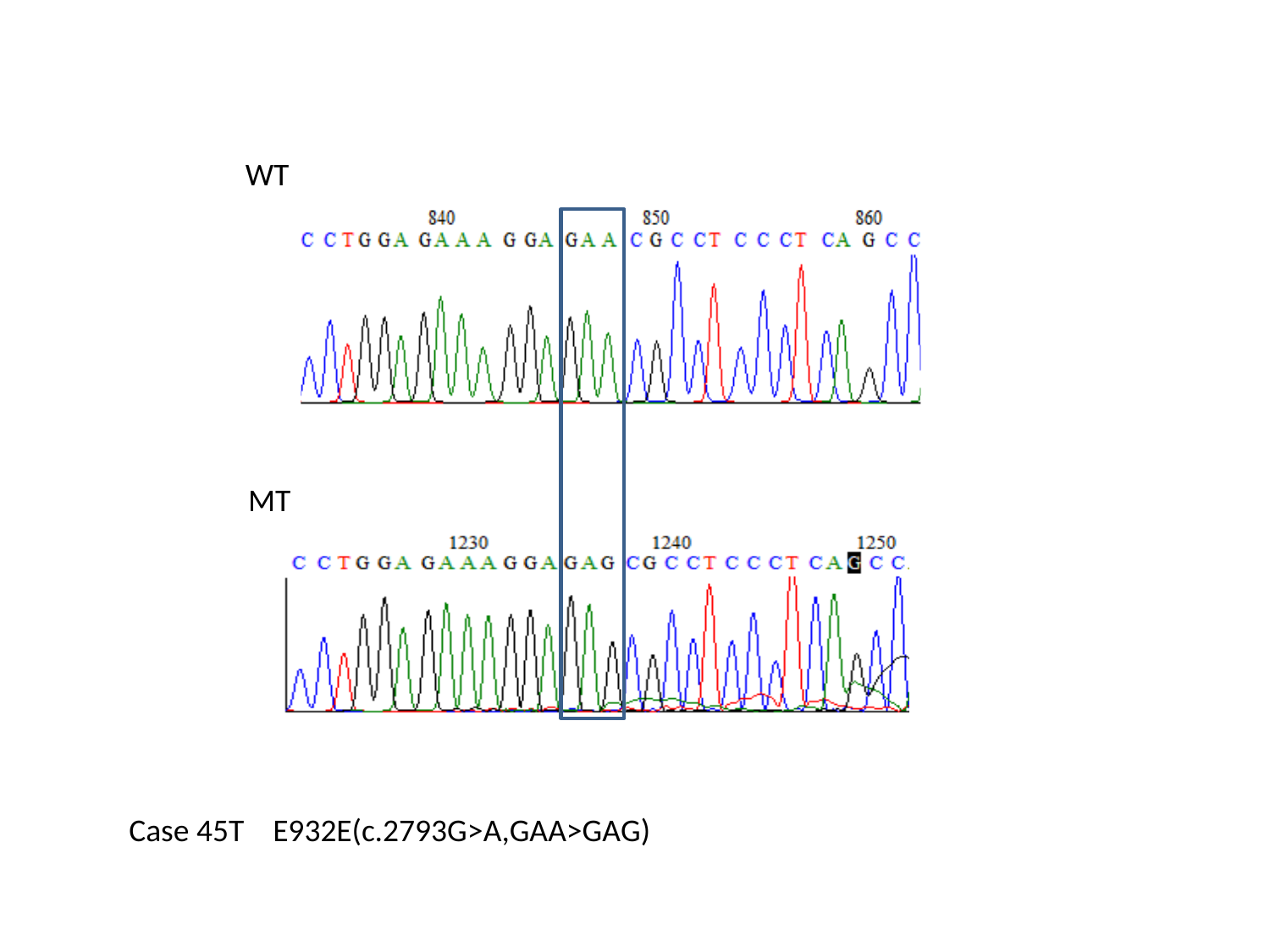

WT
MT
Case 45T E932E(c.2793G>A,GAA>GAG)
